# Glossary of terms: A shared understanding of the common terms used to describe psychological trauma, version 3.0

**DOI:** 10.24095/hpcdp.43.10/11.09

**Published:** 2023-11

**Authors:** Alexandra Heber, Valerie Testa, Dianne Groll, Kimberly Ritchie, Linna Tam-Seto, Ashlee Mulligan, Emily Sullo, Amber Schick, Elizabeth Bose, Yasaman Jabbari, Jillian Lopes, R. Nicholas Carleton

**Affiliations:** 1 Veterans Affairs Canada, Ottawa, Ontario, Canada; 2 Canadian Institute for Pandemic Health Education and Response (CIPHER), Regina, Saskatchewan, Canada; 3 Department of Psychiatry and Behavioural Neurosciences, McMaster University, Hamilton, Ontario, Canada; 4 Centre for Mental Health and Wellbeing, Public Health Agency of Canada, Ottawa, Ontario, Canada; 5 Department of Psychiatry, Queen’s University, Kingston, Ontario, Canada; 6 Trent/Fleming School of Nursing, Trent University, Peterborough, Ontario, Canada; 7 Department of Occupational Science and Occupational Therapy, Temerty Faculty of Medicine, University of Toronto, Toronto, Ontario, Canada; 8 Atlas Institute for Veterans and Families, The Royal, Ottawa, Ontario, Canada; 9 Department of Psychology, Neuroscience, and Behaviour, McMaster University, Hamilton, Ontario, Canada; 10 Canadian Institute for Public Safety Research and Treatment (CIPSRT), University of Regina, Regina, Saskatchewan, Canada; 11 Anxiety and Illness Behaviours Laboratory, Department of Psychology, University of Regina, Regina, Saskatchewan, Canada

## Preamble

***Background***

Posttraumatic stress disorder (PTSD) is a potentially disabling condition that is now widely recognized as a public health issue.^1^ PTSD and other mental disorders are more common among first responders and other public safety personnel (PSP), Canadian Armed Forces members, Veterans, and health care personnel than among the general population in Canada.^2^ The COVID-19 pandemic increased our reliance on the health care workers and public safety personnel and took an additional toll on their physical, mental, and emotional health.^3^

In 2018, the *Federal Framework on Post-Traumatic Stress Disorder Act (Act C-211)* was passed by the Parliament of Canada to address the “clear need for persons who have served as first responders, firefighters, military personnel, corrections officers and members of the RCMP to receive direct and timely access to PTSD support.”^4^ *Act C-211* is the only national government legislation, to the best of our knowledge, that acknowledges the importance of work-related risk factors that disproportionately affect the development of PTSD in certain occupations.

The Act also called for the creation of a federal framework on PTSD. The Public Health Agency of Canada (PHAC) was mandated to lead the development of this framework.^4^

Early in the development of the *Federal Framework on Posttraumatic Stress Disorder: Recognition, collaboration and support*,^1^ it was recognized that there was a need to identify relevant terminology in order to mobilize knowledge and increase understanding of the nature of PTSD. With PHAC and other national partners, the Chief Psychiatrist of Veterans Affairs Canada (VAC) and the Scientific Director of the Canadian Institute for Public Safety Research and Treatment (CIPSRT) led the development of the first version of the glossary of terms in 2019, for a national meeting organized to develop the Federal Framework on PTSD.

***Prevalence of PTSD***

The first nationwide research investigating the proportion of Canadian PSP reporting symptom clusters consistent with various mental disorders found that 23.2% of the total sample screened positive for PTSD.^2^ By contrast, estimates of the prevalence of PTSD in the general population range from 1.1% to 3.5%.^2^ Prior to the COVID-19 pandemic, pan-Canadian research, using the Carleton et al.^1^ online survey and the same psychiatric scales, reported rates of PTSD similar to those found in PSP among the regulated nurses (i.e. registered nurses, licensed practical nurses, registered practical nurses, registered psychiatric nurses, nurse practitioners) who participated in the survey, with almost one-quarter of respondents (23%) screening positive for current symptoms consistent with PTSD.^5^-^7^ Recent research suggests that since the start of the COVID-19 pandemic, rates of PTSD have increased among both health care providers and PSP.^3^

***Evolution of the glossary of terms***

Discussions around PTSD and related mental health conditions often lack a common language for people living and working in different contexts. This glossary is intended to bridge those gaps by promoting a shared understanding of many of the common terms used to describe mental health challenges arising from exposure to potentially psychologically traumatic events and stressors. Assembling and defining the terms that describe mental health and mental health conditions is a substantial challenge. No universally accepted list works for every person and every situation. In addition, words used to describe mental health and mental illness have different meanings for different people in different contexts. As the fields of psychiatry, psychology, mental health, and behavioural health are ever-changing, the glossary is a “living” document that will be revised over time to reflect new information and understanding. The list of key experts, partners, and stakeholders contributing to each iteration will also continue to evolve.

The first version of the glossary was planned and developed to facilitate open discourse among the many academics, researchers, clinicians, policy experts, non-governmental organization members, PSP, serving and veteran Canadian Armed Forces members, and people with lived experience of PTSD who attended the 2019 National Conference on PTSD; to assist in the development of the *Federal Framework on Posttraumatic Stress Disorder;*^1^ and to address key priority areas in *Supporting Canada’s Public Safety Personnel: An action plan on post-traumatic stress injuries*.^8^ A revised version of the glossary was released the same year, based on feedback on the first version, and version 2.1, with minor editorial revisions, was posted on the CIPSRT website in 2020. 

Development of version 3.0 of the glossary began in 2021. Given the pandemic’s far-reaching effects on all facets of the health care and public safety workforce, the focus of this glossary has been expanded to include health care professionals as well as serving Canadian Armed Forces members and Veterans.

The glossary focuses on PTSD and closely related terms; the senior authors team has no intention of minimizing the importance of other mental health challenges (e.g. depression, anxiety, psychosis, substance misuse, and suicidality) that can arise from exposure to one or more potentially psychologically traumatic events.

***Evolution of the terms***

Clinicians use words with care to describe and summarize complex signs, symptoms, and diagnoses, and to connect patients and clients with the treatments most likely to help them. Careful use of language also helps researchers understand the need for and ways to develop better tools for the assessment, treatment, diagnosis, and mitigation of mental health problems. The *Diagnostic and Statistical Manual of Mental Disorders* (DSM 5-TR)^9^ and the *International Classification of Diseases, 11^th^ Revision *(ICD-11)^10^ each provide widely used criteria for diagnosing mental disorders.

The language used to describe various aspects of mental health and mental illness is continually being refined. Approximately 20 terms have been added to version 3.0 of the glossary. Some of the terms have not been unanimously agreed upon by contributors, and debate about definitions is ongoing; regardless, in all cases we have provided the most balanced and collaborative definition possible. Only four terms are diagnostic categories in either the DSM-5-TR or ICD-11: burnout (only included in ICD-11), acute stress disorder (ASD), posttraumatic stress disorder (PTSD), and complex PTSD (C-PTSD). Many related terms are in use, with varying degrees of support and shared understanding.

The glossary identifies terms that are frequently used colloquially or that could be subsumed within the definitions of other terms. These terms are included as part of an effort to shift towards more accurate and less stigmatizing language. In some cases, alternatives have been suggested, or explicitly recommended, because of historically inappropriate use, stigma, or confusion.

As mentioned previously, many terms have different meanings for people in fields other than health care. In addition, cultural factors, including values, preferences, clinical experience, and research results, shape how we think about mental health and mental illness. For example, “injury” is increasingly replacing “disorder” to describe some mental health conditions. On the one hand, this shift may help diminish the stigma associated with “disorder” by aligning the symptoms or experiences more closely with physical injuries, which are often considered more “acceptable” or “honourable.” On the other hand, “disorder” communicates important information to health care providers about a person’s condition, functional limitations, and the optimal lines of treatment.

The senior authors and contributors wrote version 3.0 to promote a shared understanding of the common terms used to describe mental health challenges arising from exposure to potentially psychologically traumatic events and stressors. We hope that this is the next step toward reducing stigma, increasing access to evidence-based care, and supporting improvements in the tools, training, and treatments intended to benefit all Canadians.

***How to use this glossary***

Terms in the current glossary are arranged alphabetically by the most commonly used synonym. Most of the terms have two complementary definitions: a “general public definition” or introductory definition, geared towards a wider readership, and an “academic definition,” which may be more detailed, or “expert.” There is some overlap between the definitions, and readers may choose to read either or both.

## Index of Terms

- Acute stress disorder (ASD)
- Adverse childhood experiences (ACEs)
- Anxiety disorder
- Burnout
- Caregiver, informal
- Caregiver burden
- Caregiver satisfaction
- Clinical anxiety
- Clinical depression
- Compassion fatigue
- Complex posttraumatic stress disorder (C-PTSD)
- Complex trauma
- Critical incident
- Cultural competency / Cultural competence
- Diagnosis / Diagnostic
- Equity, diversity, and inclusion (EDI)
- Evidence-based activities
- Evidence-based medicine (EBM)
- First responder
- Gender-based violence
- Health
- Informal caregiver
- Institutional betrayal / Sanctuary trauma
- Interpersonal violence
- Intersectionality
- LGBT purge
- Mental disorder
- Mental health
- Mental health condition / Mental health challenge
- Mental health injury / Psychological injury
- Mental illness
- Military sexual trauma (MST)
- Moral injury / Moral distress / Moral dilemma
- Occupational stress injury / Organizational stress injury
- Operational stress injury (OSI)
- Panic attack
- Peer support
- People with lived experience [of a mental disorder or mental health condition] / Experts by experience [of a mental disorder or mental health condition]
- Posttraumatic growth (PTG)
- Posttraumatic psychological stress / Posttraumatic stress / Posttraumatic stress syndrome / Posttraumatic stress symptoms / Psychological stress / Psychological trauma / Psychologically traumatic stress / Traumatic stress
- Posttraumatic stress (PTS)
- Posttraumatic stress disorder (PTSD)
- Posttraumatic stress injury (PTSI)
- Potentially psychologically traumatic event (PPTE) / Psychologically traumatic event / Potentially psychologically traumatic stressor / Psychologically traumatic stressor (PTS) / Traumatic event / Traumatic stressor
- Provider
- Psychological trauma / Psychologically traumatic injury
- Public safety personnel (PSP)
- Recovery
- Resilience / Resiliency
- Sanctuary trauma
- Secondary traumatic stress
- Social support
- Stigma
- Stress / Stressor / Stressful event
- Trauma / Traumatic injury
- Tri-services
- Vicarious trauma
- Vicarious traumatic stress
- Well-being (wellbeing)
- Wellness
- Wellness check

## Terms

**Acute stress disorder (ASD)**

***General public definition***

- *Acute stress disorder* (ASD) is listed as a diagnosis in the DSM-5-TR and ICD-11.
- ASD describes a collection of feelings, behaviours, and experiencesthat can occur in the first month after a person is exposed to a potentially psychologically traumatic event (e.g. actual or threatened death, serious injury, or sexual violence). The exposure can occur in many ways. For details of exposures that may be associated with an ASD diagnosis, see the “Potentially psychologically traumatic event (PPTE)” section.
- ASD reactions can include:
  - immediate feelings of terror, panic, anxiety, rage, or sickness when you are exposed to a sound, sight, or smell that reminds an individual of the event
  - vivid and intrusive memories of the event that feel as if the event is happening again (“flashbacks”)
  - nightmares and disturbed sleep
  - not remembering the event (amnesia), or feeling emotionally numb
  - avoiding places, people, or circumstances that remind an individual of the event
  - being hyperalert to threat or danger, and
  - feeling that things are unreal or that you are living in a dream (depersonalization or derealization).
- ASD may develop into posttraumatic stress disorder after one month.
- ASD is diagnosed if there is no other physical or mental health condition that better explains the person’s condition.

***Academic definition***

- *Acute stress disorder *(ASD) is currently listed as a diagnosis in both the DSM-5-TR and ICD-11.
- ASD is mental disorder that can occur after exposure to actual or threatened death, serious injury, sexual violence, or to multiple concomitant exposures. Details of exposures that may be associated with a diagnosis for ASD are provided in the “Potentially psychologically traumatic events (PPTE)” section.
- Similar to posttraumatic stress disorder (PTSD) symptoms, ASD symptoms may include (but are not limited to):
  - Recurrent involuntary memories on exposure to reminders of the event
  - An altered sense of reality, as if the event is recurring or the patient / client is living in a dream (i.e. “flashbacks”)
  - Amnesia for important aspects of the event
  - Exaggerated startle response and other forms of hyperarousal on exposure to reminders of the event
  - Intense or prolonged psychological distress in response to cues that resemble an aspect of the event
  - Irritability and angry outbursts
  - Hypervigilance
  - Sleep disturbance and nightmares
  - Problems concentrating
  - Inability to feel positive emotions, or persistent feelings of numbness or detachment, and
  - Avoidance of any reminders of the psychologically traumatic event (including memories, thoughts, feelings, or people, places, activities that are reminders of the event).
- ASD may be diagnosed if the signs and symptoms have lasted for more than two days but less than one month and cause clinically significant distress or impairment in social, occupational, or other important areas of functioning.
- ASD may be diagnosed if the symptoms and signs are not better explained by another mental or physical health condition or the effects of a substance.
- ASD can evolve into posttraumatic stress disorder or other mental disorders.

**Adverse childhood experiences (ACEs)**

***General public definition***

- *Adverse childhood experiences* (ACEs) are currently not listed as a diagnosis in the DSM-5-TR or ICD-11.
- ACEs are potentially psychologically traumatic events in a child’s family or social life that disrupt the child’s health and cause harm or distress.
- ACEs include:
  - emotional or physical neglect
  - emotional, physical, or sexual abuse, or
  - violence in the household.
- ACEs can make children vulnerable to later life mental health problems or disorders.

***Academic definition***

- *Adverse childhood experiences* (ACEs) are currently not listed as a diagnosis in either the DSM-5-TR or ICD-11.
- “Adverse childhood experiences are defined operationally as childhood events, varying in severity and often chronic, occurring in a child’s family or social environment that cause harm and distress, thereby disrupting the child’s physical or psychological health and development.”^11^
- Examples include (but are not limited to):
  - exposure to emotional, physical, or sexual abuse
  - emotional or physical neglect
  - exposure to intimate partner violence, or
  - dysfunction within the household (e.g. exposure to parental separation or a family member with a history of mental disorder, substance misuse, incarceration, suicide attempt, or death by suicide).

**Anxiety disorder**

***General public definition***

- *Anxiety disorder *is currently not listed as a diagnosis in the DSM-5-TR or ICD-11, although anxiety disorders are a class of psychiatric disorders in the DSM-5-TR.
- Anxiety is part of the body’s early warning system of anticipated danger. Anxiety is a normal reaction when you are confronted by something frightening or threatening. Common features of anxiety are worry, nervousness, restlessness, sweating, rapid heart rate, muscle tension, irritability, trouble concentrating, and feelings of dread.
- Sometimes people experience symptoms of anxiety when there is no obvious threat. This may be because the person has internal feelings and concerns that trigger the same physical and psychological responses as a threat. If these anxiety symptoms are severe or long-lasting, they may be part of an anxiety disorder. Panic disorder, social phobia, or generalized anxiety disorder are some of the diagnoses that are classified as anxiety disorders.
- Anxiety disorders can be successfully treated with psychotherapy and/or medication.

***Academic definition***

- *Anxiety disorder* is currently not listed as a diagnosis in either the DSM-5-TR or ICD-11, although anxiety disorders are a class of psychiatric disorders listed in the DSM-5-TR.
- Anxiety disorders appear to be caused by the cognitive, emotional, and physiological overactivation of the fear response, which results in anxiety symptoms and disruption to normal psychosocial functioning. According to the DSM-5-TR, several diagnoses that share common features of anxiety, excessive fear, and behavioural disturbance are classified as anxiety disorders:
  - Separation anxiety
  - Selective mutism
  - Specific phobia
  - Social phobia
  - Panic disorder
  - Agoraphobia
  - Generalized anxiety disorder
  - Substance/medication-induced anxiety disorder, and
  - Anxiety disorder due to another medical condition
- Symptoms of anxiety disorders are often aversive and disruptive to normal functioning. They commonly include feelings of worry, nervousness, restlessness, rapid heart rate, muscle tension, irritability, hyperventilation, sleep disruption, feelings of dread, and difficulties with attention and concentration.

**Burnout**

***General public definition***

- *Burnout* is currently not listed as a medical or psychiatric diagnosis in the DSM-5-TR or ICD-11, although it is listed as a condition in the ICD-11.
- *Burnout* is an occupational occurrence that an employee experiences because of stress in the workplace, particularly organizational stress (e.g. ongoing conflict with supervisors or colleagues; working large amounts of overtime; having insufficient break time at work; feeling that the employer does not recognize the employee’s effort and contributions).
- *Burnout* may be the condition underlying overwhelming exhaustion, cynicism or feeling detached from the job, or feeling ineffective or resentful for not getting rewarded.

***Academic definition***

- *Burnout* is currently described as an “occupational phenomenon” in the ICD-11, but it is not listed as a medical or psychiatric diagnosis.
- The World Health Organization describes *burnout* as largely precipitated by the workplace or organization having deleterious effects on the individuals working at that workplace or organization.
- The ICD-11 describes *burnout* as “a syndrome conceptualized as resulting from chronic workplace stress that has not been successfully managed.... Burn-out refers specifically to phenomena in the occupational context and should not be applied to describe experiences in other areas of life.”^10^
- Burnout is a “psychological syndrome in response to chronic interpersonal stressors on the job. The three key features of this response are: an overwhelming exhaustion, feelings of cynicism and detachment from the job, and a sense of ineffectiveness and lack of accomplishment.”^12^
- Burnout can involve a negative change in reaction to others, including inappropriate attitudes toward coworkers, irritability, loss of idealism, and withdrawal.^13^
- Perceived “high caseloads, lack of control or influence over agency policies and procedures, unfairness in organizational structure and discipline, low peer and supervisory support, and poor agency and on-the-job training”^16^^,p.59^ have been identified as organizational factors underlying burnout.^14^-^16^
- *Burnout* is distinct from *compassion fatigue, vicarious traumatic stress, secondary traumatic stress* and *vicarious trauma*, as it is not necessarily related to exposures to potentially psychologically traumatic events, complex trauma, or adverse childhood experiences.

**Caregiver, informal**

***General public definition***

- *Informal caregivers* typically provide care to a family member or friend without being paid for the work.
- The care may be provided every day or occasionally, and for a short or a long period of time.

***Academic definition***

- *Informal caregivers* typically provide unpaid care daily, or occasionally, and for a short or long time.
- The informal caregiver’s involvement or role is primarily characterized by a personal relationship, rather than by a professional relationship with monetary compensation.

**Caregiver burden**

***General public definition***

- *Caregiver burden* means the additional negative effects, on the caregiver, of caring for someone.
- These negative effects can include less available time for social activities, leisure time, or work, and financial problems.
- The caregiver may also experience physical, psychological, social, and emotional impacts, and feel fatigued, depressed, anxious, helpless, and socially isolated.^17^

***Academic definition***

- *Caregiver burden* is divided into two categories:
  - *Objective caregiver burden* refers to the negative effects of the care recipient’s illness on the caregiver because of the demands placed on the caregiver’s time for work and social and leisure activities, and/or financial burden.
  - *Subjective caregiver burden* refers to the physical, psychological, social, and emotional impact of caregiving, such as feelings of fatigue, depression, anxiety, helplessness, and social isolation.^17^

**Caregiver satisfaction**

***General public definition***

- *Caregiver satisfaction* refers to the positive effects of providing care—personal satisfaction, feeling needed, useful, and good about oneself, and/or closer to the person receiving the care.

***Academic definition***

- *Caregiver satisfaction* is the term most commonly used to refer to the positive aspects of caregiving.
- Possible positive effects of caregiving include a strengthening of the relationship with the care recipient, personal growth, increased self-esteem, and a sense of purpose.

**Clinical anxiety**

***General public definition***

- *Clinical anxiety* is currently not listed as a diagnosis in the DSM-5-TR or ICD-11.
- The term *clinical anxiety* is used colloquially to describe anxiety symptoms severe enough to require formal medical or mental health treatment.
- *Clinical anxiety* symptoms can include intense worry, nervousness, restlessness, rapid heart rate, muscle tension, irritability, trouble concentrating, and feelings of worry or dread, or physical changes such as increased blood pressure.
- See the “Anxiety disorder” section for more information.

***Academic definition***

- *Clinical anxiety* is currently not listed as a diagnosis in the DSM-5-TR or ICD-11.
- The term *clinical anxiety* is used colloquially to describe anxiety symptoms severe enough to require formal medical or mental health treatment. It can refer to a number of different diagnoses (e.g. generalized anxiety disorder, panic disorder).
- Refer to the “Anxiety disorder” section for more information.

**Clinical depression**

***General public definition***

- *Clinical depression* is currently not listed as a diagnosis in the DSM-5-TR or ICD-11.
- The term *clinical depression* may be used colloquially to describe symptoms of a major depressive episode that requires formal medical treatment.
- A major depressive episode is typically a period of at least several weeks of feeling sad or numb, having low mood, lack of interest in usual activities or relationships, difficulty concentrating, poor sleep, feelings of hopelessness, and sometimes, suicidal thoughts. Without treatment, a major depressive episode can last from several months to a year or longer.
- Clinical depression may indicate the need for one or more formal treatments that may include medication, psychotherapy, and assessment for suicidality.

***Academic definition***

- *Clinical depression* is currently not listed as a diagnosis in the DSM-5-TR or ICD-11.
- The term *clinical depression* is used colloquially to describe symptoms of a major depressive episode that requires formal medical treatment.
- A major depressive episode is typically a period of at least several weeks of feeling sad or numb, having low mood, lack of interest in usual activities and/or relationships, difficulty concentrating, poor sleep, feelings of hopelessness, and sometimes, suicidal thoughts. Without treatment, a major depressive episode can last from several months to a year or more.
- Clinical depression may signify the need for one or more formal treatments including medication, psychotherapy, and assessment for suicidality.

**Compassion fatigue**

***General public definition***

- *Compassion fatigue* is currently not listed as a diagnosis in the DSM-5-TR or ICD-11.
- *Compassion fatigue* is an emerging condition, meaning that it has not been well studied and is not yet fully defined. It is a type of mental health condition that caregivers can experience.
- *Compassion fatigue *refers to the stress of caring for patients or clients, family members, or others.
- *Compassion fatigue *can create a sense of helplessness, confusion, and at times, a loss of empathy for others.
- People who are experiencing *compassion fatigue* can feel emotionally isolated from colleagues, family members, and other important people in their lives.

***Academic definition***

- *Compassion fatigue* is currently not listed as a diagnosis in either the DSM-5-TR or ICD-11.
- *Compassion fatigue* is an emerging condition that has not yet been well studied, elaborated, understood, or researched, and the term remains controversial. *Compassion fatigue* appears to be a series of adverse psychological reactions to providing care to others. The adverse reactions appear related to the emotional toll and stress of caring for others.
- *Compassion fatigue* is often described as occurring among those who provide psychological care to patients or clients who have experienced one or more potentially psychologically traumatic event.^18^
- Compassion fatigue can include a sense of helplessness or confusion and a loss of empathy toward the person who is being treated or helped, as well as a feeling of emotional isolation from colleagues, family, and other social supports.
- Compassion fatigue can occur as a result of a single exposure or an accumulation of exposures to patients’ or clients’ detailed descriptions of their psychologically traumatic experiences.
- *Compassion fatigue* is sometimes associated with, or conflated with, *secondary traumatic stress or vicarious trauma*. *Compassion fatigue* is generally considered to be distinct from *burnout*.

**Complex posttraumatic stress disorder (C-PTSD)**

***General public definition***

- *Complex posttraumatic stress disorder *(C-PTSD) is listed as a diagnosis in the ICD-11, but not in the DSM-5-TR.
- C-PTSD is a type of posttraumatic stress disorder that results from experiencing repeated, severe psychologically traumatic events that the person cannot escape, such as adverse childhood experiences or situations of forced captivity and torture.
- People with C-PTSD have a profound loss or absence of a sense of identity and difficulty controlling their emotions. They are often involved in unstable relationships as adults, and often have patterns of impulsive choices, feelings of emotional instability, and behavioural problems. Sometimes they experience chronic unhappiness, suicidal thoughts, and suicide attempts.
- There is evidence that people with C-PTSD are likely to experience dissociative symptoms such as periods of amnesia where they cannot remember their actions, have feelings of unreality or of losing time, or experience themselves as having fragmented or multiple personalities.
- *Complex trauma* is often used to refer to C-PTSD.

***Academic definition***

- *Complex posttraumatic stress disorder *(C-PTSD) is listed as a diagnosis in the ICD-11, but not in the DSM-5-TR.
- C-PTSD meets the DSM-5-TR diagnostic criteria for *posttraumatic stress disorder*, with additional criteria, as specified in the ICD-11.
- C-PTSD is distinguished from *posttraumatic stress disorder *mainly by the protracted and interpersonal nature of the potentially psychologically traumatic event(s) (e.g. chronic childhood sexual abuse by a trusted person such as a parent, or an extended period of captivity and torture, including incarceration in a concentration camp); the subsequent distortions of the person’s sense of self and core personal and social identity; and the significant emotional dysregulation. *Posttraumatic stress disorder* is more typically associated with a discrete psychologically traumatic event or a series of psychologically traumatic events that usually occur after childhood.^19^,^20^
- Dr. Judith Herman, one of the pioneers investigating childhood psychological trauma, has described C-PTSD in detail.

**Complex trauma**

***General public definition***

- *Complex trauma* is currently not listed as a diagnosis in the DSM-5-TR or ICD-11.
- *Complex trauma* is a psychological injury that results from severe types of potentially psychologically traumatic events.
- *Complex trauma* is often used to refer to *complex posttraumatic stress disorder* (C-PTSD).
- See the section on “Complex posttraumatic stress disorder (C-PTSD)” for more information.

***Academic definition***

- *Complex trauma* is currently not listed as a diagnosis in the DSM-5-TR or ICD-11.
- *Complex trauma* describes a psychological injury resulting from exposure to multiple psychologically traumatic events or a single prolonged psychologically traumatic event, particularly when the event was difficult to escape, such as repeated childhood sexual or physical abuse, prolonged domestic violence, torture, enslavement, or genocide campaigns.^21^-^23^
- Refer to the section on “Complex posttraumatic stress disorder (C-PTSD)” for more information.

**Critical incident**

***General public definition***

- *Critical incident* is currently not listed as a diagnosis in the DSM-5-TR or ICD-11.
- The term *critical incident* is most often used to describe a potentially psychologically traumatic event experienced by public safety personnel (PSP) that evokes unusually strong emotional reactions.
- A critical incident may occur when a person is overwhelmed by the scope, severity, personal connection to, or degree of exposure to a potentially psychologically traumatic event.
- The term* critical incident *is often used interchangeably with *potentially psychologically traumatic event* (PPTE) / *psychologically traumatic event / potentially psychologically traumatic stressor / psychologically traumatic stressor (PTS) / traumatic event / traumatic stressor or trauma / traumatic injury*.

***Academic definition***

- *Critical incident *is currently not listed as a diagnosis in either the DSM-5-TR or the ICD-11.
- The term *critical incident *is most commonly used by public safety personnel (PSP) to describe a potentially psychologically traumatic event (PPTE).
- Critical incidents are thought to involve experiencing unusually strong emotional reactions that have the potential to interfere with a person’s ability to function either at the scene of a PPTE or following the PPTE. Critical incidents can involve “all physical custody (arrests), all vehicle and foot pursuits, all dispatched code responses (emergency), all motor vehicle accidents that require physical work and all calls which present an active threat to life and/or property.”^24^
- The term *critical incident* used to be used to distinguish common exposures in the line of duty from exposures thought more likely to cause psychological problems.

**Cultural competency / Cultural competence**

***General public definition***

- The term *cultural competency*, or *cultural competence*, is used to describe the need for mental health professionals to better understand the work experiences and workplace environment of the patients or clients (such as military members or public safety personnel [PSP]) who are at risk for exposure to psychologically traumatic events while doing their jobs.
- *Cultural competency *is the ability of health care providers to work respectfully, and more effectively, with patients and clients through understanding and challenging their own biases, and actively educating themselves on the workplace environment and culture of those they work with.
- PSP define *cultural competency* as a feeling of being understood, that “they get us.”
- In the public safety and military environments, *cultural competency* often refers to sector-specific knowledge of the workplace, work-related terminology, and the types of potentially psychologically traumatic events to which these workers are exposed.

***Academic definition***

- The term *cultural competency*, or *cultural competence*, is used to describe the need for mental health professionals to better understand the work experiences and workplace environment of their patients and clients (e.g. military members or public safety personnel [PSP]) who are at risk for psychologically traumatic exposures arising from experiences in their working environment while doing their jobs.
- *Cultural competency* is the ability of health care providers (and, by extension, any professional) to work respectfully and more effectively with people from diverse cultures, including workplace cultures, while understanding and challenging their own biases and actively educating themselves on the workplace culture(s) of the people they work with.
- PSP define cultural competency as a feeling of being understood, that “they get us.”
- A culturally competent mental health provider conducts all interactions with patients and clients in a manner that recognizes, affirms, values, and preserves the dignity of the individual and their experiences.

**Diagnosis / Diagnostic**

***General public definition***

- A *diagnosis* is an explanation, made by a qualified health care professional within their scope of practice, of an individual’s mental or physical health condition or conditions.
- The *Diagnostic and Statistical Manual of Mental Disorders* (DSM) and the *International Classification of Diseases* (ICD) provide diagnostic criteria for mental disorders.

***Academic definition***

- A *diagnosis* is the conclusion of a process to determine the nature of a disease, disorder, or health condition, including distinguishing it from other possible diseases, disorders, or health conditions.
- A *diagnosis* refers to the justifiable conclusion made by a regulated health care professional within their scope of practice, usually using the criteria outlined in the *Diagnostic and Statistical Manual of Mental Disorders* (DSM) or *International Classification of Diseases* (ICD).
- The most widely accepted criteria for making diagnoses of mental disorders are those listed in the DSM or the ICD.
- In psychiatry and psychology, a *diagnosis* is the process of explaining a person’s state of mental health by conducting an examination of the relevant history and current mental and physical status of the person, and from this information, drawing one or more conclusions about the nature of the health condition(s) or disease(s).
- A diagnosis of a mental disorder should not be made without considering a differential diagnosis of physical disorders and other mental disorders that could explain the person’s current state of health. Physical disorders may occur concurrently with mental disorders.

**Equity, diversity, and inclusion (EDI)**

***General public definition***

- *Equity, diversity, and inclusion *(EDI) describe three closely linked values that promote belonging and an environment of fair treatment for all individuals, including those who might otherwise be excluded or marginalized.

***Academic definition***

- *Equity* is defined as the fair, impartial, and unbiased treatment of people.
- *Diversity* refers to the range of different characteristics or the demographic heterogeneity of a group of people.
- *Inclusion* is the creation of an environment where there is equal access to opportunities and resources for people who might otherwise be excluded or marginalized.
- Equity, diversity, and inclusion (EDI) fosters an environment where diversity is accepted and acknowledged, where there is equal opportunity to fully participate, and where disparities between people and groups are reduced to ensure equal chance for success.
- EDI refers to how societies create equal opportunity for all people, or workplaces create equal opportunity for all employees, regardless of their differences.
- EDI balances accepting differences and supporting equal opportunity for success, through acknowledging and reducing any negative impact of these differences.

**Evidence-based activities**

***General public definition***

- Terms such as *evidence-based* or *evidence-informed activities, evidence-based leadership, evidence-based clinical practice, or evidence-informed practice are derived from the concept of evidence-based medicine* (EBM).
- The degree to which each of these terms emphasizes the three components of EBM—the clinician’s expertise, the best current clinical evidence, and the client or patient’s wishes and concerns—depends on the context in which the term is being used and the understanding and goals of the person using the term.

***Academic definition***

- *Evidence-based or evidence-informed activities, evidence-based leadership, evidence-based clinical practice, or evidence-informed practice* are derived from the concept of *evidence-based medicine* (EBM).
- The degree to which each of these terms emphasizes the three components of EBM—the clinician’s expertise, the best current clinical evidence, and the patient’s wishes and concerns—depends on the context in which the term is being used and the understanding and goals of the person using the term.

**Evidence-based medicine (EBM)**

***General public definition***

- *Evidence-based medicine* (EBM) is the routine integration into every health care encounter of three things:
  - The experience and expertise of the clinician
  - The best currently available clinical evidence obtained from systematic research (which is almost always published in peer-reviewed journals), and
  - The wishes and concerns of the patient or client.
- EBM is now the accepted standard for clinical practice by all physicians and legitimate health care providers.
- When you first go to a new health care provider, ask them if they practise evidence-based medicine (to make sure that they know what it is).

***Academic definition***

- The term *evidence-based medicine* (EBM) was coined by Dr.Gordon Guyatt of McMaster University in 1992.^25^
- EBM is the routine integration into every health care encounter of the following:
  - The experience and expertise of the clinician
  - The best currently available clinical evidence obtained from systematic research, and
  - The wishes and concerns of the patient or client.
- EBM makes decision-making in clinical practice more structured and objective by combining current population-based research findings with the practitioner’s clinical expertise in diagnosis and patient or client care and their compassionate awareness of the individual patient or client’s predicaments, rights, and preferences.^26^-^29^
- EBM is the accepted standard for legitimate clinical practice, and people should be encouraged to ask a new health care provider if they practise evidence-based medicine (to ensure that the provider knows what this means).

**First responder**

***General public definition***

- A *first responder* is a professional with specialized training who arrives at the scene of an emergency to provide immediate medical or evacuation help. The emergency could be a motor accident, a natural disaster, or a terrorist attack.
- The definition of *first responder* continues to evolve and may change.
- Historically, first responders have included firefighters, paramedics, and police officers.
- Many people also consider other trained members of organizations that provide professional assistance during an emergency (e.g. military personnel and public safety communicators such as 911 dispatchers) to be first responders.

***Academic definition***

- A* first responder* is an officially mandated person with specialized training who arrives at the scene of an emergency, such as a motor accident, natural disaster, or terrorist attack, to provide medical or evacuation assistance.
- This definition continues to evolve.
- Historically, first responders have often included firefighters, paramedics, and police officers.
- Other trained members of organizations who provide professional assistance during an emergency (e.g. military personnel and public safety communicators such as 911 dispatchers) may also be considered as first responders.

**Gender-based violence**

***General public definition***

- *Gender-based violence* is currently not listed as a diagnosis in the DSM-5-TR or ICD-11.
- *Gender-based violence* refers toany type of harmful behaviour against a person or group of people because of their sex, gender, sexual orientation, or gender identity.

***Academic definition***

- *Gender-based violence* is currently not listed as a diagnosis in the DSM-5-TR or ICD-11.
- The Government of Canada defines gender-based violence as “violence based on gender norms and unequal power dynamics, perpetrated against someone based on their gender, gender expression, gender identity, or perceived gender. It takes many forms, including physical, economic, sexual, as well as emotional (psychological) abuse.”^30^

**Health**

***General public definition***

- *Health* describes how you are feeling and functioning physically, mentally, socially, and in some definitions, spiritually.
- *Health* can also describe how you view your own “physical, mental, social, and spiritual functioning”^31^ or how other people view your “physical, mental, social, and spiritual functioning.”^31^

***Academic definition***

- *Health* describes the physical, mental, social, and (according to some) spiritual functioning of a person, and is on a continuum from poor to good.^32^-^35^
- *Health* can be described subjectively, for example, when a person describes their own physical functioning, psychological well-being, or health-related quality of life.
- *Health* also can be described objectively, for example, when a family member or health care professional makes observations about another person’s physical functioning, psychological well-being, or health-related quality of life.

**Informal caregiver**

See the “Caregiver, informal” section.

**Institutional betrayal / Sanctuary trauma**

***General public definition***

- *Institutional betrayal* and *sanctuary trauma* are currently not listed as diagnoses in the DSM-5-TR or ICD-11.
- *Institutional betrayal* is occasionally associated with moral injury or burnout.
- *Institutional betrayal* focuses on an organization failing to prevent or respond to wrongdoings within the organization.
- *Sanctuary trauma* focuses on the experiences of a person who was treated poorly or abused in an organization that the person believed was a safe place.
- *Sanctuary trauma *is usually used to describe the experiences of those living with the effects of military sexual trauma. See the “Military sexual trauma (MST)” section for more information.

***Academic definition***

- Neither *institutional betrayal* nor *sanctuary trauma* are currently listed as diagnoses in the DSM-5-TR or ICD-11.
- *Institutional betrayal*, a term introduced by Dr. Jennifer J. Freyd,^36^ refers to institutions (i.e. a governing body or organization) causing harm to those who depend on those institutions (e.g. employees, residents). This term is commonly used to describe experiences of moral injury or burnout where the institution (and those in charge) fail to intervene and prevent or respond supportively to challenges within the institution, where an individual expects some degree of fair treatment or protection.^36^-^38^
- Dr. Steven Silver^39^ defines *sanctuary trauma* as a psychologically traumatic event that “occurs when an individual who suffered a severe stressor next encounters what was expected to be a supportive and protective environment and discovers only more trauma.”
- *Institutional betrayal* and *sanctuary trauma* both involve actions that bring up feelings of vulnerability, helplessness, fear, and shame, and both occur in environments or institutions that we expected to be safe—such as families, schools, workplaces, governments, hospitals, the military, and religious institutions.
- The difference between the two concepts is that *institutional betrayal* focuses on the failure of the institution, whereas *sanctuary trauma *focuses on the experience of the individual who expected the institution to be a safe place or sanctuary.

**Interpersonal violence**

***General public definition***

- *Interpersonal violence* is currently not listed as a diagnosis in the DSM-5-TR or ICD-11.
- *Interpersonal violence* is the harmful physical and psychological behaviour by a person or group of people toward another person.
- *Interpersonal violence* is a type of potentially psychologically traumatic event or stressor. Interpersonal violence can contribute to mental health conditions in either the person causing the harm or the person who is harmed.
- Examples of interpersonal violence include intimate partner violence, elder abuse, and workplace violence.

***Academic definition***

- *Interpersonal violence* is currently not listed as a diagnosis in either the DSM-5-TR or ICD-11.
- *Interpersonal violence* occurs when an individual causes physical or psychological injury to another individual, through one or more behaviours. Interpersonal violence includes, but is not limited to, child maltreatment, intimate partner violence, elder abuse, assault by strangers, as well as violence to do with property crimes or in the workplace or other institutions.
- Potentially psychologically traumatic events that involve interpersonal violence may cause more severe or complex mental health conditions (including mental disorders) due to interpersonal betrayal and attachment disruption. Other potentially psychologically traumatic events (e.g. natural disasters, structure fires) can also lead to similar severity and complexity of mental health conditions. However, the personal and relational nature of interpersonal violence often leads to more complexmental health conditions.

**Intersectionality**

***General public definition***

- People are not a simple and one-dimensional group. Numerous intersecting identity factors impact how each of us understands and experiences the world around us.
- Identity factors include ethnicity, religion, age, physical and cognitive ability, sex, gender identity, sexual orientation, and socioeconomic status.
- Intersectionality recognizes the multiple aspects of an individual’s identity that influence their experiences.

***Academic definition***

- The term *intersectionality* was coined in 1989 by scholar and civil rights activist Kimberl Crenshaw^40^ to explain how race interacts with sex and/or gender and other factors to create barriers for Black women. Today, intersectionality is considered more broadly as a framework for understanding how aspects of a person’s identity combine to create different modes of discrimination and privilege.
- Intersectionality is an approach to identity that recognizes that different identity categories can intersect and coexist in the same individual in a way that creates a qualitatively different experience when compared to any of the individual characteristics involved.

**LGBT purge**

***General public definition***

- Between the 1950s and the 1990s, lesbian, gay, bisexual, and transgender (LGBT) members of the Canadian Armed Forces, the Royal Canadian Mounted Police, and theCanadian federal public service were systematically discriminated against, harassed, and often fired as a matter of policy and sanctioned practice, in what came to be known as the LGBT Purge.
- The LGBT Purge affected more than 9000 Canadian citizens. In 2016, survivors of the LGBT Purge launched a nationwide class action lawsuit against the Government of Canada. The class action settlement reached in 2018 included a financial settlement of $145 million.^41^

***Academic definition***

- For more than 40 years from the 1950s, lesbian, gay, bisexual, and transgender (LGBT) members of the Canadian Armed Forces, the Royal Canadian Mounted Police, and the Canadian federal public service were systematically discriminated against, harassed, and often fired as a matter of policy and sanctioned practice.^41^,^42^
- In what came to be known as the LGBT Purge, LGBT or suspected LGBT personnel were followed, interrogated, arrested, abused, and as a result, traumatized.
- The Canadian government’s LGBT Purge affected more than 9000 Canadian citizens. In 2016, survivors of the LGBT Purge launched a nationwide class action lawsuit against the Government of Canada. A settlement reached in 2018 included a financial settlement of $145 million dollars.^41^,^42^

**Mental disorder**

***General public definition***

- A *mental disorder* is a type of mental health condition that meets the criteria for a diagnosis published in the DSM-5-TR or ICD-11 or other equivalent revisions.
- A health care practitioner will diagnose a mental disorder if the person’s history and clinical examination meet diagnostic criteria that best explain the person’s current condition.
- Mental disorders are often caused by mechanisms other than exposure to a potentially psychologically traumatic event. Mental disorders may be caused by the interactions of many factors—family and childhood history, recent experiences or stressors, genetics, biology, socioeconomic factors, physical health problems, and physical environmental factors.
- Examples of mental disorders include major depressive disorder, bipolar disorder, generalized anxiety disorder, personality disorders, and schizophrenia.
- You can have more than one mental disorder at a time, and these disorders may interact with each other.
- Usual responses to common stressors, such as the death of a loved one, usual workplace pressures, or living with a physical health condition or chronic pain are not mental disorders—unless these responses continue for a long period of time, cause very high levels of distress, or cause symptoms from which the person cannot recover.

***Academic definition***

- A *mental disorder* is a clinically significant disturbance in an individual’s cognition, emotion regulation, and/or behaviour that reflects dysfunction in the psychological, biological, or developmental processes underlying mental functioning, where the person’s condition meets DSM-5-TR or ICD-11 diagnostic criteria.
- A mental disorder is associated with significant distress in social, occupational, or other activities.
- Causes of mental disorders are thought to be multiple and interlinked, not linear, and related to various combinations of physically or psychologically traumatic events, genetics, biology, diet, socioeconomic factors, physical health conditions, physical environmental factors, and other factors.
- The symptoms and signs of a mental disorder are not better explained by another mental disorder, a physical health condition or the effects of a substance.
- Common culturally consistent responses to a stressor or loss that do not meet accepted diagnostic criteria, such as the death of a loved one, are not mental disorders.
- Socially deviant behaviour (e.g. political or sexual) and conflicts that are primarily between the individual and society are not necessarily mental disorders, unless the behaviour(s) results from a dysfunction in the individual caused by a mental or physical health condition.

**Mental health**

***General public definition***

- *Mental* refers to thoughts, feelings, emotions, and related brain functioning.
- *Mental health* exists on a continuum from poor to good. In good mental health, a person:
  - understands themselves and their abilities
  - copes well with normal stress
  - experiences good feelings from their interactions and relationships with others
  - is able to work or function well in their usual activities, and
  - contributes to their family and/or community.
- You can be diagnosed with a mental disorder but still cope well and have good mental health. On the other hand, you can have poor mental health without having a diagnosis of a mental disorder.
- Mental healthis influenced by a variety of personal and social factors and stresses.

***Academic definition***

- *Mental* refers to thoughts (thought content), feelings (emotions), and related brain functioning.
- *Mental health* exists on a continuum from poor to good, with good mental health being a state of psychological well-being. Good mental health generally enables people to function in their lives and usual activities, realize their potential, cope with the normal stresses of life, and contribute to their families and/or community.
- *Mental disorder* lies on a different continuum, on a scale from mild to severe.^43^ A person can have good mental health even if they have a mental illness or a mental disorder. More commonly, a person can have poor mental health but still not meet the diagnostic criteria for any specific mental disorder described in the DSM-5-TR or ICD-11.
- Mental health is influenced by a wide variety of personal, social, and economic determinants.

**Mental health condition / Mental health challenge**

***General public definition***

- *Mental health condition*, or *mental health challenge*, is currently not listed as a diagnosis in the DSM-5-TR or ICD-11.
- A mental health condition refers to any state of poor mental health or mental illness.
- This term can be used to describe normal reactions to everyday stressors as well as mental disorders.
- Some people use the term *mental health challenge* instead of *mental health condition *to try to reduce the stigma associated with mental illness.

***Academic definition***

- *Mental health condition*, or *mental health challenge*, is currently not listed as a diagnosis in the DSM-5-TR or ICD-11.
- This is a broad term that includes mental disorders, mental illness, and undiagnosed symptoms that might be explained by a diagnosis of a mental disorder. Mental health conditions often are caused by mechanisms other than exposures to one or more potentially psychologically traumatic events. Examples include major depressive disorder, bipolar disorder, generalized anxiety disorders, personality disorders, and schizophrenia.
- Mental health conditions may also include states of poor mental health that do not meet DSM-5-TR or ICD-11 diagnostic criteria for a mental disorder, for example, culturally consistent responses to common stressors.
- Some people prefer to use the term “mental health challenge” rather than “mental health condition” in an effort to reduce the stigma associated with mental illness.

**Mental health injury / Psychological injury**

***General public definition***

- *Mental health injury* and *psychological injury* are currently not listed as diagnoses in either the DSM-5-TR or ICD-11.
- *Mental health injury* and *psychological injury* may also be used as different names for a mental health condition, including a mental disorder, especially when the condition is caused by exposure to one or more potentially psychologically traumatic events.
- The word “injury” is used when describing mental disorders or conditions to try to reduce the stigma associated with mental illness. The terms *mental health injury *and *psychological injury* were coined by members of the Canadian Armed Forces to describe posttraumatic stress disorder as a mental injury that results from engagement in military operations and as “honourable” as physical injury (see the “Operational stress injury (OSI)” section).

***Academic definition***

- *Mental health injury* and *psychological injury* are currently not listed as diagnoses in the DSM-5-TR or ICD-11.
- These are terms describing a mental health condition, including a mental disorder, especially when the mental health condition is caused by exposure to one or more potentially psychologically traumatic events.
- The word “injury” is used when describing mental disorders or conditions in an attempt to reduce the stigma associated with mental illness. The terms *mental health injury* and *psychological injury* were initially coined by members of the Canadian Armed Forces to describe posttraumatic stress disorder (PTSD) as a mental injury that results from engagement in military operations and no less “honourable” than physical injury (refer to the “Operational stress injury (OSI)” section).

**Mental illness**

***General public definition***

- *Mental illness* refers to emotions, behaviours, and difficulties in thinking that affect a person’s ability to clearly understand the real world and what is happening to them and around them. These emotions, behaviours, and difficulties are generally the signs and symptoms of the mental illness.
- The signs and symptoms of a mental illness can range from mild to severe.
- These signs and symptoms can usually be diagnosed as a particular kind of mental illness, as outlined in the DSM-5-TR or the ICD 11.
- Mental illness is often accompanied by distress and decreased functioning in social, occupational, or other activities of daily life.

***Academic definition***

- *Mental illness* refers to mental disorders, diagnosed or as yet undiagnosed.
- Mental illness ranges in severity from mild to severe.
- Mental illness is associated with significant distress in social, occupational, or other activities of daily living.

**Military sexual trauma (MST)**

***General public and academic definition***

- MST is currently not listed as a diagnosis in the DSM-5-TR or ICD-11.
- MST refers to any sexual or sexualized activity that occurs without the person’s consent, during their service as a member of the CAF, and the physically or psychologically traumatic impacts of this activity on the affected person. The spectrum of MST can vary from small impact to severe disorders.
- Examples of sexual or sexualized activities without the person’s consent or where the person is unable to consent include (but are not limited to):
  - Taking part in sexual activities because of coercion or threat (such as threats to a person’s physical safety, reputation, or career progression, or threats of other negative treatment, if the person refuses to comply)
  - Any coercive situation where expectation of, participation in, or tolerance of, unwanted sexual experiences is used as a basis for work assignment or promotion decisions
  - Any situation involving comments, unwanted touching, grabbing, or sexual advances, including hazing activities or rituals
  - Sexual contact or activities while sleeping, unconscious, or any other circumstance where the person’s capacity to consent is impaired by drugs or alcohol
  - Sexualized comments or displays of pornographic or demeaning materials in the workplace
  - Repeated unwelcome requests for a sexual relationship
  - Witnessing any of the examples of sexual or sexualized activities in this list
  - Any unwanted sexual activity or display that creates a hostile, intimidating, or offensive work environment.
- Examples of MST impacts on the affected person include (but are not limited to):
  - Disturbed sleep or nightmares
  - Feeling sad or depressed
  - Disturbing memories of re-experiencing the event
  - Difficulty feeling safe
  - Feeling numb or without emotion
  - Feeling guilt or shame, anger or rage
  - Problems in work (such as reduced productivity, conflict with coworkers)
  - Problems in intimate relationships, and difficulties parenting
  - Problems with alcohol or drugs
  - Physical injuries or pain conditions, and
  - Reluctance to report for duty or to wear their uniform.

**Moral injury / Moral distress / Moral dilemma**

- The terms *moral injury*, *moral distress*, and *moral dilemma* fall along a continuum. *Moral injury* is usually understood as the most severe and debilitating experience, *moral dilemma* as the least, and *moral distress* as somewhere between the two.

***General public definition***

- *Moral injury, moral distress*, and *moral dilemma* are currently not listed as diagnoses in the DSM-5-TR or ICD-11.
- The meanings of these terms are evolving and may change.
- During potentially psychologically traumatic events or other unusually stressful situations, people may carry out, witness, or fail to prevent events that go against their moral beliefs and expectations.^44^
- A moral injury can occur in response to doing something or witnessing behaviours or acts that go against a person’s values and moral beliefs. Events that cause moral injury can be:
  - acts of commission (what someone has done)
  - acts of omission (what someone has failed to do), or
  - acts of betrayal.

***Academic definition***

- *Moral injury, moral distress*, and *moral dilemma* are currently not listed as diagnoses in either the DSM-5-TR or ICD-11. These are evolving concepts.
- *Moral injury* describes the psychological, social, and spiritual distress, harm, or impairment that results from experiencing a violation of deeply held morals, ethics, or values.
- When an event has the potential to cause moral injury, it is called a *potentially morally injurious event* (PMIE).
- The development of moral injury following a PMIE may parallel the development of posttraumatic stress disorder following a potentially psychologically traumatic event.
- Moral injury may impact the following domains:
  - emotional (increased feelings of guilt, shame, anger)
  - spiritual (loss of spiritual or religious beliefs, loss of sense of life’s purpose)
  - self (alterations in identity, self-perception)
  - moral (changes in moral appraisals of self and others), and
  - relational (difficulty maintaining existing relationships or fostering new ones).
- During potentially psychologically traumatic events or other unusually stressful circumstances, people may perpetrate, fail to prevent, or witness events that contravene their deeply held moral beliefs and expectations.^38^
- “When [individuals do] something that goes against their beliefs, this is … referred to as an act of commission, and when they fail to do something in line with their beliefs, that is … an act of omission. Individuals may experience betrayal from leadership, others in positions of power, or peers, [which] can result in adverse outcomes.”^45^
- “Moral injury is the distressing psychological, behavioural, social, and sometimes spiritual aftermath of exposure” to acts of commission, omission, or betrayal.^46^
- “A moral injury can occur in response to acting in a way or witnessing behaviours that go against an individual’s values and moral beliefs.”^47^
- “In order for moral injury to occur, the individual must feel like a transgression occurred and that they or someone else crossed a line with respect to their moral beliefs. Guilt, shame, disgust and anger are some of the hallmark reactions of moral injury.”^47^

**Occupational stress injury / Organizational stress injury**

***General public definition***

- *Occupational stress injury*, or *organizational stress injury*, is currently not listed as a diagnosis in the DSM-5-TR or ICD-11.
- *Occupational stress injury* and *organizational stress injury* are derived from *operational stress injury* to convey the idea of stress occurring in organizations or work situations outside of a military operational environment.
- These are new and emerging definitions that are as yet neither well defined nor clear and they may change.
- These terms may be mistakenly used interchangeably with *operational stress injury* (OSI); always be precise when referring to allthese terms.
- Only use the acronym “OSI” when you are referring to *operational stress injury*.

***Academic definition***

- *Occupational stress injury*, or *organizational stress injury*, is currently not listed as a diagnosis in the DSM-5-TR or ICD-11.
- These are evolving concepts. One view is that occupational stressors can be sorted into *operational stressors* and *organizational stressors*; however, the terms are emerging, and the definitions remain unclear and may change.
- Staff shortages, lack of training on new equipment, lack of appropriate resources, inconsistent leadership styles, and a perceived lack of coworkers’ and leaders’ support are examples of organizational stressors.

**Operational stress injury (OSI)**

***General public definition***

- *Operational stress injury* (OSI) is currently not listed as a diagnosis in the DSM-5-TR or ICD-11.
- This definition was developed by members of the Canadian Armed Forces.
- OSI refers to any mental disorder or other mental health condition resulting from operational duties performed while serving in the Canadian Armed Forces.
- This term is often used by public safety personnel to describe mental health problems that may result from performing their assigned duties, for example, a police officer’s anxiety symptoms after witnessing a shooting incident or a potentially psychologically traumatic death.
- *Operational stress injury* (OSI) is often mistakenly used interchangeably with *organizational stress injury* or *occupational stress injury*; it is important, therefore, to be specific when referring to each of these three terms.

***Academic definition***

- *Operational stress injury* (OSI) is currently not listed as a diagnosis in either the DSM-5-TR or the ICD-11.
- OSI refers to any mental disorderor othermental health condition resulting from operational duties performed while serving in the Canadian Armed Forces.
- The term and definition were developed by the same team of military personnel who designed the Canadian Armed Forces peer support initiative, Operational Stress Injury Social Support (OSISS), as part of a broad effort to destigmatize the mental health symptoms that military members can develop after exposure to one or more potentially psychologically traumatic events.
- The decision to use “injury” in the term was deliberate, to help people perceive mental injuries as on par with the physical injuries that military personnel sometimes sustain, which tend to be perceived as “honourable” injuries.
- Generally, the operational stressors associated with an operational stress injury are potentially psychologically traumatic events.
- OSI is used to describe a broad range of mental disorders that may not meet DSM or ICD criteria for mental disorders but nevertheless are debilitating, cause distress, and interfere with daily functioning in social, work, and family life. These include:
  - Posttraumatic stress disorder
  - Anxiety disorders
  - Depressive disorders
  - Substance use disorders, and
  - Other mental health conditions.
- The term is often used by public safety personnel to describe mental health problems that may result from performing their assigned duties.
- OSI is sometimes mistakenly used synonymously with *organizational stress injury* or *occupational stress injury*, but the literature defines these differently.
- Only OSI has been defined and used with any regularity in the mental health community.

**Panic attack**

***General public definition***

- *Panic attack* is currently not a diagnosis in the DSM-5-TR or ICD-11. It is a group of symptoms that can be part of a diagnosis of a panic disorder.
- Panic attacks can occur “out of the blue,” for apparently no reason, or can be triggered by a feared situation.
- Panic attack symptoms include shortness of breath, racing heart, chest pain or pressure, sweating, shakiness, nausea, dizziness, numbness/tingling sensations, feelings of unreality, and fear of losing control or of dying.
- Symptoms usually start abruptly and intensify quickly.
- Panic attacks are extremely frightening and can cause a great deal of disability if they occur repeatedly and are not treated.
- Treatment with both psychotherapy and medication is very effective.

***Academic definition***

- *Panic attack* is currently not listed as a diagnosis in either the DSM-5-TR or the ICD-11. It is a characteristic group of symptoms that can be part of a diagnosis of a panic disorder.
- The DSM-5-TR defines a panic attack as a sudden rush of intense fear and discomfort that peaks rapidly and is associated with characteristic physical and cognitive symptoms. The DSM-5-TR specifies a minimum of four symptoms, which may include:
  - physiological sensations (palpitations, sweating, shakiness, shortness of breath, feelings of choking, chest pain, nausea, dizziness, numbness/tingling sensations, feelings of derealization/depersonalization), or
  - cognitions (fear of losing control or fear of dying).
- If fewer than four symptoms are present, the episode is classified as a *limited-symptom panic attack*.
- A panic attack is not a mental disorder on its own.If it meets a number of criteria, it can be classified as a panic disorder, which is a discrete mental disorder. Panic attacks may occur in the context of any mental or medical disorder, although they are most commonly associated with anxiety disorders. When panic attacks are present, they are noted as a specifier to the diagnosed disorder, with the exception of panic disorder, where the presence of panic attacks is a central criterion for diagnosis.
- The ICD-11 describes a panic attack as an episode of “intense fear,” characterized by a rush of physical symptoms (e.g. palpitations, chest pain, feelings of choking, dizziness, feelings of unreality), and/or cognitive symptoms that include fear of dying, losing control, or “going mad.”^10^ The ICD-11 notes that panic attacks are a feature of panic disorder.
- Panic attacks may occur unexpectedly, or “out of the blue.” Alternatively, they may occur because of exposure to a feared situation or trigger (e.g. an individual with a snake phobia may develop a panic attack in response to seeing a picture of a snake).
- Untreated, recurring panic attacks can be extremely disabling.
- Treatment of panic attacks with both medication and psychotherapy is very effective.

**Peer support**

***General public definition***

- *Peer support* is a supportive, recovery-oriented relationship between individuals who have had or have the same experience, that is, they have a shared lived or living experience.
- Peer support can offer social, emotional, spiritual, and instrumental support to promote a person’s well-being and path to recovery from mental health problems.
- Shared lived and living experiences can be the basis for a peer support relationship. These may be physical and/or mental health experiences, experiences of problematic workplaces, housing, or finances, or shared experiences of grief and loss.
- Peer support can be delivered one-to-one or by a group, and some peer support is a combination of both.
- To provide peers with safe and effective support, the expertise gained by the peer supporter through their lived experience is often supplemented with training (in basic counselling techniques and theory, as well as fields related to the shared experience).
- Examples of peer support are Alcoholics Anonymous (AA) and Operational Stress Injury Social Support (OSISS).

***Academic definition***

- The literature defines *peer support* in different ways; at its core, peer support is a supportive, recovery-oriented relationship between individuals that is based on perceived shared characteristics and shared lived and living experiences, such as experiencing posttraumatic stress disorder.^48^-^50^
- The shared lived and living experiences that can be the basis for a peer support relationship include physical and/or mental health experiences, experiences of problematic housing, finances, or workplaces, and experiences of grief and loss.
- Peer support can be delivered one-to-one or in a group, and some peer support is a combination of both.
- Peer support offers recovery-oriented social, emotional, and spiritual support, which is frequently coupled with instrumental support (e.g. psychoeducation, referral support, system navigation).^51^
- A peer who offers peer support may be called a *peer supporter*, a *lived-experience peer*, a *peer support worker*, a *well-being responder*, or a *paraprofessional*, among other terms.^48^
- A peer supporter’s proclivity for empathetic understanding of others in a similar or shared lived or living experience provides an automatic credibility with peer(s) seeking support.
- There are currently no broadly accepted or evidence-based standards related to peer support training; however, the knowledge and expertise of this experience is enhanced when supplemented with training (often in basic counselling techniques and theory, as well as fields related to the shared experience).^52^-^55^
- Examples of peer support are Alcoholics Anonymous (AA) and Operational Stress Injury Social Support (OSISS).

**People with lived experience [of a mental disorder or mental health condition] / Experts by experience [of a mental disorder or mental health condition]**

***General public definition***

- *People with lived experience* or *experts by experience* of a mental disorder or mental health condition are living with, have lived with, or are recovering from a mental disorder or mental health condition.

***Academic definition***

- *People with lived experience* or *experts by experience* of a mental disorder or mental health condition are living with, have lived with, or are recovering from a mental disorder or mental health condition.
- These terms may also include people who were exposed to one or more potentially psychologically traumatic event.
- These terms may also include the family members and other supporting persons who are engaged with people who are living with, have lived with, or are recovering from a mental disorder or mental health condition or exposure to one or more potentially psychologically traumatic events.

**Posttraumatic growth (PTG)**

***General public definition***

- *Posttraumatic growth* (PTG) is currently not listed as a diagnosis in the DSM-5-TR or ICD-11.
- PTG refers to the positive personal changes that may result from a person’s struggle to manage the consequences of being exposed to one or more potentially psychologically traumatic events.
- The positive personal changes of PTG may include a new appreciation for life and future possibilities, a newfound sense of personal strength, improved relationships with others (e.g. a new focus on helping others), and spiritual or existential change.
- PTG is not merely bouncing back to pre-trauma levels of functioning, but includes positive growth beyond pre-trauma levels of functioning and well-being.

***Academic definition***

- *Posttraumatic growth* (PTG) is currently not listed as a diagnosis in either the DSM-5-TR or the ICD-11.
- PTG refers to positive personal changes that may result from an individual working to cope with the psychological consequences of exposure to one or more potentially psychologically traumatic events.
- Major dimensions of PTG include enhancement of relationships (e.g. increased empathy, humility, and altruism); changes in self-perception (e.g. of personal resiliency, strength); increased acceptance of vulnerability and limitations; changes in life philosophy (e.g. re-evaluating what’s important); and spiritual or existential change.^56^,^57^
- PTG involves not merely reverting to pre-trauma levels of functioning, but experiencing positive growth beyond those pre-trauma levels of functioning.
- PTG and posttraumatic stress can occur simultaneously.

**Posttraumatic psychological stress / Posttraumatic stress / Posttraumatic stress syndrome / Posttraumatic stress symptoms / Psychological stress / Psychological trauma / Psychologically traumatic stress / Traumatic stress**

***General public definition***

- *Posttraumatic psychological stress, posttraumatic stress, posttraumatic stress syndrome, posttraumatic stress symptoms, psychological stress, psychological trauma, psychologically traumatic stress, and traumatic stress* are currently not listed as diagnoses in the DSM-5-TR or ICD-11.
- These terms are all used to describe possible outcomes from exposure to one or more potentially psychologically traumatic events (PPTEs).
- The most commonly used of these terms is *posttraumatic stress symptoms* (PTSS).

***Academic definition***

- *Posttraumatic psychological stress, posttraumatic stress, posttraumatic stress syndrome, posttraumatic stress symptoms, psychological stress, psychological trauma, psychologically traumatic stress, and traumatic stress* are currently not listed as diagnoses in the DSM-5-TR or ICD-11.
- These are colloquial terms used to describe possible outcomes from exposure to one or more potentially psychologically traumatic events (PPTEs). These terms may be used to describe evidence of a diagnosis of PTSD.

**Posttraumatic stress (PTS)**

***General public definition***

- *Posttraumatic stress *(PTS) is currently not listed as a diagnosis in the DSM-5-TR or ICD-11.
- PTS has been used to refer to stress resulting from exposure to one or more potentially psychologically traumatic events. PTS has also been used to refer to *posttraumatic stress disorder* and to other mental health conditions that can follow exposure to one or more potentially psychologically traumatic events and interfere with daily functioning in social, work, or family activities.
- PTS is often used interchangeably with other terms. Using more specific terms is often helpful.

***Academic definition***

- *Posttraumatic stress* (PTS) is currently not listed as a diagnosis in the DSM-5-TR or ICD-11.
- PTS may refer to posttraumatic stress disorder, but it has also been used to refer to mental health conditions with some features of posttraumatic stress disorder that nevertheless do not meet the diagnostic criteria for *posttraumatic stress disorder*. In either case, PTS still interferes with daily functioning in social, work, or family activities.
- PTS can develop soon after exposure to a single potentially psychologically traumatic event or progressively, over time, with cumulative exposures.
- PTS does not refer to reactions to stressful events or significant life changes other than potentially psychologically traumatic events, and does not refer to normal reactions to common stressors.
- PTS is a colloquial term, often used interchangeably with the diagnostic term *posttraumatic stress disorder *(PTSD). The appropriate use of* posttraumatic stress disorder* or PTSD is more accurate and less confusing.

**Posttraumatic stress disorder (PTSD)**

***General public definition***

- *Posttraumatic stress disorder* (PTSD) is listed as a diagnosis in both the DSM-5-TR and ICD-11.
- PTSD is the collection of feelings, behaviours, and experiences that can occur after a person is exposed to a potentially psychologically traumatic event (e.g. actual or threatened death, serious injury, or sexual violence). The exposure can occur in many ways. For details of exposures that may be associated with a PTSD diagnosis, see the “Potentially psychologically traumatic event (PPTE)” section.
- PTSD reactions can include:
  - immediate feelings of terror, panic, anxiety, rage, or sickness when exposed to a sound, sight, or smell that is a reminder of the event
  - vivid and intrusive memories of the event, which can sometimes feel as if the event is happening again (“flashbacks”)
  - nightmares and disturbed sleep
  - not remembering the event (amnesia) or feeling emotionally numb
  - avoiding places, people, or circumstances that are reminders of the event
  - being hyperalert to threat or danger, and
  - feeling that things are unreal or that you are living in a dream (depersonalization or derealization).
- PTSD symptoms can also include having negative thoughts and difficulty feeling emotionally connected to family, friends, or other people who are important to the person.
- A person may be diagnosed with PTSD if the experiences and symptoms last for more than one month and cause significant distress or affect how they function in social, occupational, or other important areas of life.
- PTSD is diagnosed if the person’s condition is not better explained by another physical or mental disorder.

***Academic definition***

- *Posttraumatic stress disorder* (PTSD) is listed as a diagnosis in both the DSM-5-TR and ICD-11.
- PTSD is a mental disorder that can occur after exposure to actual or threatened death, serious injury, or sexual violence or to multiple concomitant exposures. Details of exposures that may be associated with a diagnosis for PTSD are provided in the “Potentially psychologically traumatic event (PPTE)” section.
- Symptoms may include (but are not limited to):
  - Recurrent involuntary memories on exposure to reminders of the event
  - An altered sense of reality, as if the event is recurring or the patient or client is living in a dream (i.e. “flashbacks”)
  - Amnesia for important aspects of the event
  - Exaggerated startle response and other forms of hyperarousal on exposure to reminders of the event
  - Intense or prolonged psychological distress in response to cues that resemble an aspect of the event
  - Irritability and angry outbursts
  - Hypervigilance
  - Sleep disturbance and nightmares
  - Problems concentrating
  - Inability to have positive emotions, or persistent feelings of numbness or detachment, and
  - Avoidance of any reminders of the psychologically traumatic event (including memories, thoughts, feelings, or people, places, activities that are reminders of the event).
- PTSD may be diagnosed if the signs and symptoms have lasted for more than one month and cause clinically significant distress or impairment in social, occupational, or other important areas of functioning.
- PTSD may be diagnosed if the symptoms and signs are not better explained by another mental or physical health condition or the effects of a substance.

**Posttraumatic stress injury (PTSI)**

***General public definition***

- *Posttraumatic stress injury* (PTSI) is currently not listed as a diagnosis in the DSM-5-TR or ICD-11.
- The term is commonly used by public safety personnel (PSP) and those who do research with PSP in Canada.
- PTSI refers to the mental health conditions that a person may experience as a result of exposure to one or more potentially psychologically traumatic events.

***Academic definition***

- *Posttraumatic stress injury* (PTSI) is currently not listed as a diagnosis in the DSM-5-TR or ICD-11, although the term is commonly used by public safety personnel (PSP) and those who do research with PSP in Canada.
- PTSI is a non-clinical term subsuming one or more mental health conditions a person may experience after exposure to one or more potentially psychologically traumatic events. These mental health conditions include symptoms related to anxiety-, mood-, or trauma-related disorders that may meet diagnostic criteria for a *generalized anxiety disorder, major depressive disorder, panic disorder*, or *posttraumatic stress disorder*.
- PTSI includes symptoms that interfere with daily functioning in social, work, or family activities. Categorizing mental health conditions and using terms such as *posttraumatic stress injuries* may be an attempt to decrease stigma.

**Potentially psychologically traumatic event (PPTE) / Psychologically traumatic event / Potentially psychologically traumatic stressor / Psychologically traumatic stressor (PTS) / Traumatic event / Traumatic stressor**

***General public definition***

- *Potentially psychologically traumatic event* (PPTE), *psychologically traumatic event, potentially psychologically traumatic stressor, psychologically traumatic stressor* (PTS), *traumatic event*, and *traumatic stressor *are currently not listed as diagnoses in the DSM-5-TR or ICD-11.
- A *psychologically traumatic event *(PTE) is a stressful event that has caused psychological trauma that may be consistent with one or more posttraumatic mental health conditions (e.g. posttraumatic stress disorder [PTSD], or panic disorder).
- The term *potentially psychologically traumatic event *(PPTE) describes events that have the potential to cause PTSD and other trauma-related mental health conditions. This term is more precise than terms like *critical incident*.
- Psychological trauma does not require a physical injury to be harmful.
- Typical examples of potentially psychologically traumatic events (PPTE) include:
  - adverse childhood experiences
  - motor vehicle accidents
  - sexual assault and other types of violence
  - unexpected violent or accidental death of a loved one or threatened death of a loved one, and
  - threat of or actual severe physical injury, experiencing military combat, natural disasters, or exposure to human remains.

***Academic definition***

- *Potentially psychologically traumatic event *(PPTE), *psychologically traumatic event, potentially psychologically traumatic stressor, psychologically traumatic stressor* (PTS), *traumatic event*, and *traumatic stressor* are currently not listed as diagnoses in either the DSM-5-TR or ICD-11.
- A *psychologically traumatic event *(PTE) is a stressful event that involves actual, perceived, or threatened death, serious injury, or sexual violence, and causes psychological trauma that may be consistent with one or more posttraumatic mental health conditions (e.g. *posttraumatic stress disorder, panic disorder*).
- *Psychological trauma *differs from* physical trauma *in that psychological trauma never requires evidence of tissue damage or dysfunction.
- Exposure to one or more *psychologically traumatic events* (PTEs) is a criterion for a diagnosis of acute stress disorder or posttraumatic stress disorder in the DSM-5-TR and ICD-11.
- The term psychologically traumatic event is preceded by the word “potentially” (i.e. “potentially psychologically traumatic event” [PPTE]) because such exposures are idiosyncratic. The descriptor “potentially” is part of an effort to underscore the importance of dynamic individual and environmental contextual factors that influence whether an event is psychologically traumatic for any given individual at any given time.
- Exposure to a PPTE can be direct, witnessed, learned about having occurred to a close family member or close friend (in such cases the event must have been violent or accidental), or through repeated or extreme exposures to aversive details.
- A PPTE may involve moral elements that require special attention (e.g. potentially morally injurious events, which can cause harm without requiring actual or threatened death, serious injury, or sexual violence).
- Not everyone exposed to a PPTE develops a posttraumatic stress injury or posttraumatic stress disorder. Pre-existing factors and concurrent or post-PPTE mental and physical injuries can affect whether a posttraumatic stress injury occurs. Examples include a prior history of traumatic event exposure, perceived helplessness during the PPTE, and perceived presence or absence of social support during and after the PPTE.
- Contemporary colloquial mental health language may use *stressor, traumatic stressor*, or *trauma* interchangeably with PPTE.
- A PPTE describes events that have the specific potential to cause posttraumatic stress disorder (PTSD) and other trauma-related mental health conditions, and is more precise than terms like *critical incident*.

**Provider**

***General public definition***

- A *health care provider* is a clinician or therapist who provides direct care to a patient or client or, sometimes, to a group of patients or clients. This individual may provide a wide variety of services.
- A health care provider can also be an organization, or the administrative staff or the governing body of a health care organization, responsible for managing or administering aspects of the care that is being delivered to patients or clients within that organization.
- All health care providers have a “duty of care” to their patients or clients; specifically, the care providers must take reasonable care to avoid anything that can cause harm or lead to the harm of those in their care.^58^
- Doctors, nurses, psychologists, social workers, nurse practitioners, physician’s assistants, medical technicians, respiratory technologists, dentists, physiotherapists, optometrists, and hospital organizations are examples of health care providers.
- Individual health care providers may work in a health care facility (e.g. a hospital, clinic, or private office) and may provide care in person, over the phone, or virtually, via the Internet.

***Academic definition***

- A *health care provider* is an individual (or a group of individuals, or an organization) that provides services from across the health care continuum to patients or clients.
- All health care providers—including hospital administrators or the hospital institution itself—have a “duty of care” to their patients or clients, that is, “the responsibility or legal obligation … to avoid acts or omissions that could likely cause harm to others.”^58^
- A health care provider may work in a health care facility such as a hospital or in a clinic or private office, and may provide care in person, by phone, or virtually, over the Internet. Or a health care provider may be a hospital or clinic administrator or the governing body of a health care institution.

**Psychological trauma / Psychologically traumatic injury**

***General public definition***

- *Psychological trauma*, or *psychologically traumatic injury*, is currently not listed as a diagnosis in the DSM-5-TR or ICD-11.
- *Psychological trauma* describes the psychological problems that may result after exposure to a psychologically traumatic event.
- Psychological trauma is an individual experience that may not present the same way for every person.
- See also the “Mental disorder,” “Mental health injury / Psychological injury,” “Posttraumatic stress disorder (PTSD),” and “Acute stress disorder (ASD)” sections.

***Academic definition***

- *Psychological trauma*, or *psychologically traumatic injury*, is currently not listed as a diagnosis in the DSM-5-TR or ICD-11.
- *Psychological trauma* is caused by exposure to a psychologically traumatic event.^22^,^59^
- The manifestations of the psychological injury, psychological trauma, or psychologically traumatic injury may be consistent with one or more mental disorders, but may also be consistent with mental health conditions for which there are no diagnostic categories in the DSM or ICD.

**Public safety personnel (PSP)**

***General public definition***

- The definition of *public safety personnel* (PSP) continues to evolve and may change.
- This is a broad term to describe the people who ensure the safety and security of the public.
- First responders can also be referred to as PSP.
- In Canada, PSP includes border services officers, serving Canadian Armed Forces members and Veterans, correctional service and parole officers, firefighters (career and volunteer), Indigenous emergency managers, operational intelligence personnel, paramedics, police officers, public safety communicators (e.g. 911 dispatchers), and search and rescue personnel.

***Academic definition***

- The definition of *public safety personnel* (PSP) continues to evolve.
- The term is currently used primarily in Canada.
- It is a broad term meant to encompass people with specialized training who are employed to ensure the safety and security of the public.
- In the 2016 report *Healthy Minds, Safe Communities*, the Standing Committee on Public Safety and National Security defined a *public safety officer* broadly “to encompass frontline personnel who ensure the safety and security of Canadians, including tri-services (fire, police, and paramedics), search and rescue personnel, correctional services officers, border services officers, operational intelligence personnel, and Indigenous emergency managers.”^60^^,p.5^
- Feedback on the term *public safety officer *indicated that many occupational groups felt excluded by the use of the word “officer” because they did not identify as belonging to military or police forces. As a result, *public safety personnel *is now used to refer to people acting in professional capacities that relate to public safety.

**Recovery**

***General public definition***

- *Recovery* is currently not listed as a diagnosis in the DSM-5-TR or ICD-11.
- *Recovery* is defined as the personalized journey to a way of living that allows a person with a physical or mental health condition to have positive mental health and good well-being.

***Academic definition***

- *Recovery* is currently not listed as a diagnosis in either the DSM-5-TR or ICD-11.
- Although there is no universally agreed-upon definition for the term *recovery*, the concept is widely endorsed.
- *Recovery* refers to the personally contextualized, self-determined journey to well-being when a person has a mental disorder, a chronic physical health condition, or chronic pain.
- Recovery is the ongoing process of change that increases a person’s well-being, includes symptom reduction, and also allows for living a meaningful life where the person has positive mental health, is hopeful and optimistic, and is participating and contributing to society, their community, or whatever the individual has defined as a worthwhile endeavour.^61^,^62^

**Resilience / Resiliency**

***General public definition***

- *Resilience*, or *resiliency*, is currently not listed as a diagnosis in the DSM-5-TR or ICD-11.
- Resilience is the long-term physical and mental tenacity that people draw on to deal with ongoing adversity.
- Sometimes referred to as “grit” or “determination,” resilience is believed to be a quality that an individual may naturally possess or develop through work and life experiences.

***Academic definition***

- *Resilience*, or *resiliency*, is currently not listed as a diagnosis in either the DSM-5-TR or ICD-11.
- The definition of this term continues to evolve. Resilience is often used along with *resistance*, *recovery*, and *reconfiguration* to describe the potential outcomes and responses to various stressors. Such stressors can be persistent or time-limited.
- The current emphasis in much of behavioural health and mental health training on enhancing resilience/y may have unintended, negative consequences. If resilience/y training is provided to an individual and they do not sustain a state of resilience/y, that individual could potentially experience blame and shame, leading to additional stigma, feelings of hopelessness, and decreased future help-seeking behaviours. This is the “down side” of over-encouraging individuals to be resilient, placing “too much blame on specific individuals for broad, systemic injustices and disadvantages, and too much responsibility on these individuals to overcome them.”^63^ Resilience/y programming has emerged through goodwill, but overall, has not been systematically monitored or evaluated.^64^

**Sanctuary trauma**

See the “Institutional betrayal / Sanctuary trauma” section.

**Secondary traumatic stress**

***General public definition***

- *Secondary traumatic stress* is currently not listed as a diagnosis in the DSM-5-TR or ICD-11.
- *Secondary traumatic stress* describes a mental health condition that results from indirect exposure to details of one or more potentially psychologically traumatic events in the course of professional duties. Such indirect exposure is also known as *vicarious trauma*.
- This is an evolving term. Secondary traumatic stress may be an occupational hazard of the helping professions, that is, people in these occupations may be at higher risk of developing secondary traumatic stress.

***Academic definition***

- *Secondary traumatic stress* is currently not listed as a diagnosis in the DSM-5-TR or ICD-11.
- *Secondary traumatic stress* is a mental health condition that may occur as a result of indirect exposure to details of one or more potentially psychologically traumatic events in the course of professional duties such as hearing the content of psychologically traumatic events experiences from patients or clients. Secondary traumatic stress is an occupational hazard of first responders, trauma care workers, and health care professionals who work with traumatized individuals.
- Secondary traumatic stress reactions can be similar to the symptoms experienced by those with posttraumatic stress disorder, and can include symptoms like reexperiencing, hypervigilance, and avoidance.
- *Secondary traumatic stress* falls within the category of “empathy-based” stress outcomes for caregivers. This category also includes constructs such as *compassion fatigue* and *vicarious trauma*. The driving cause of *secondary traumatic stress* is an empathic relationship that is the conduit for exposure to details of one or more potentially psychologically traumatic events.

**Social support**

***General public definition***

- At its simplest, you can think of *social support *as the extent to which you feel yourself supported by others.
- People have social support networks of different sizes. Social support also includes various groups or networks, including people from work, family, and friends from different parts of a person’s life.
- We sometimes pick from our respective social networks the people who will “be there for us,” and as a result, those who help us experience better health and well-being.

***Academic definition***

- At its simplest, *social support* can be conceptualized as the extent to which people experience themselves as supported by others.
- Perception of social support comprises a key aspect of the social support construct that is positively associated with emotional health and well-being, and negatively associated with depression and other negative health outcomes.
- Social support is defined by the perception of being supported by others, and able to rely upon others for emotional and/or instrumental support, or the sense that others will “be there for us” when needed.
- Variables that are inversely associated with social support, be it conceptually and/or empirically, include loneliness, social isolation, a perception of burdening others, and social hopelessness; those variables that are positively or directly associated with social support include acceptance, connectedness, positive rapport with or identification with others, and “mattering.”
- Measures and methods for assessing the presence, nature, and degree of social support vary by the aspect of social support being examined.
- Interventions to promote social support have varied in complexity, from in-person or online peer-support groups and psychotherapy groups, to individual psychological interventions designed to promote and strengthen social support and camaraderie, and reduce or prevent negative psychological symptoms and outcomes.

**Stigma**

***General public definition***

- *Stigma* is currently not listed as a diagnosis in the DSM-5-TR or ICD-11.
- *Stigma* is a set of highly negative ideas and beliefs that society has about something. To be stigmatized means to be symbolically marked as a disgrace.
- Mental illnesses are among the most highly stigmatized conditions in society. When a health condition is stigmatized, the person experiences not only the symptoms of the condition itself, but also the social rejection, disapproval, and the shame that the stigmatization creates.
- Because mental illnesses often have no physical manifestations, that is, they are “invisible,” people are not readily confronted by their attitudes and beliefs about them. This invisibility also encourages those who are experiencing mental illnesses and mental health conditions to continue to hide and remain silent about their condition, which adds to their feelings of shame and can prolong the stigmatized beliefs and attitudes about the condition.
- In the past few years, public health campaigns on mental illness and mental health, as well as the accounts of public figures who have been open about their mental health struggles, have helped to decrease the stigma surrounding mental illness.
- There are four types of mental health stigma:
  - Structural stigma is when organizational policies and practices are unfair to people with mental disorders.^65^
  - Public or interpersonal stigma is when the public think or act negatively toward people with a mental disorders.^66^
  - Self-stigma occurs when people with a mental disorder believe the negative public views and apply these views to themselves.^67^
  - Stigma-by-association is when those close to or related to the stigmatized group (such as family, friends, or mental health providers) are also stigmatized.^68^

***Academic definition***

- *Stigma* is currently not listed as a diagnosis in either the DSM-5-TR or ICD-11.
- “In ancient Greece, citizens pricked marks on their slaves using a pointed instrument, both to demonstrate ownership and to signify that such individuals were unfit for citizenship. The ancient Greek word for prick is ‘stig,’ and the resulting mark, a ‘stigma.’ In modern times, stigma is understood as an invisible mark that signifies social disapproval and rejection.”^69^ Nowadays, to be stigmatized means to be symbolically marked as a disgrace.
- “Stigma is deeply discrediting and isolating, and it causes feelings of guilt, shame, and inferiority and a compulsion to hide or a wish for concealment.”69
- Mental illnesses are among the most highly stigmatized conditions in our society. When a health condition is stigmatized, the person experiences not only the symptoms of the condition, but also the social rejection, disapproval, and shame that the stigmatization creates.
- A broad definition of stigma that includes multiple interrelated elements most closely fits the day-to-day experiences of people with mental health conditions.
  1. The first element involves the identification and labelling of socially salient differences (i.e. a mental illness).
  2. Next, the label is linked to one or more negative stereotypes, for example, that people with PTSD are violent and unpredictable.
  3. Once labelled, people are placed into a distinct social category and seen as separate from the norm (“us versus them”). They are no longer considered unique individuals but part of a homogeneous group sharing undesirable characteristics.
  4. Once categorized in this way, stigmatized people experience discrimination and loss of status, which leads to social inequities in many areas of life.^70^
- There are four main types of stigma:
  - Structural stigma reflects the accumulated policies and practices of organizations that intentionally or unintentionally create social inequities for people with mental disorders.^65^
  - Public or interpersonal stigma reflects the negative attitudes and behaviours of members of the general public toward people with mental disorders.^66^
  - Self-stigma occurs when people with mental disorders and other mental health conditions internalize the stigmatizing views of the public and apply them to themselves. As a result, they may experience diminished worth, lowered self-esteem, disempowerment, poor morale, and worse quality of life.^67^
  - Stigma-by-association occurs when negative public reactions are transferred to those close to the stigmatized individual, including the services and supports, such as mental health providers, mental health programs, and mental health research.^68^

**Stress / Stressor / Stressful event**

***General public definition***

- *Stress, stressor, and stressful event *are currently not listed as diagnoses in the DSM-5-TR or ICD-11.
- *Stress* means the way you feel, or look like you feel, when you are affected by a stressor.
- Stress is a common experience, and some stress can be positive if it leads to growth and adaptation; however, stress can result in psychological trauma.
- When you are experiencing a stressful event, you are being impacted by one or more *stressors* that are putting pressure on you physically or mentally. If the experience is severe enough, the stress may result in a psychological trauma that can lead to a mental health condition, such as posttraumatic stress disorder.
- *Stress, stressor,andstressful event* are often used interchangeably to refer to a potentially psychologically traumatic event or an adverse childhood experience.

***Academic definition***

- *Stress, stressor, and stressful event* are currently not listed as diagnoses in either the DSM-5-TR or ICD-11.
- *Stress* describes the experience a person has while being impacted by one or more stressors. Stress is often characterized by psychological distress and physiological changes (e.g. increased heart rate, shallow breathing, muscle tension).
- Stress is a common experience and some stress can be beneficial if the stress leads to growth and adaptation; however, stress can also result in psychological trauma.
- A stressor is a physical, radiological, biological, socioeconomic, or psychological force that acts upon a person during events such as a motor vehicle accident, loss of an important relationship, loss of employment, confronting an attacker, dealing with financial loss, or adverse childhood experiences.
- During a psychologically stressful event, a stressor causes an emotional experience, which is accompanied by predictable biochemical, physiological, and behavioural changes.
- The word *stress* is often used synonymously, sometimes mistakenly and confusingly, when people are referring to traumatic stress, psychological trauma, a mental health condition, or a mental disorder associated with experiencing a stressful event.
- *Stress, stressor, and stressful event* are often used interchangeably to refer to a potentially psychologically traumatic event or an adverse childhood experience.

**Trauma / Traumatic injury**

***General public definition***

- *Trauma and traumatic injury *are currently not listed as diagnoses in the DSM-5-TR or ICD-11.
- A *trauma* is something that causes physical, emotional, spiritual, or psychological harm.
- In the mental health context, *trauma* is a person’s experience during an event that is so distressing to them that it overwhelms them emotionally; psychological trauma can be the cause of mental disorders such as posttraumatic stress disorder.
- Psychologically stressful experiences, however, are not necessarily traumatic.

***Academic definition***

- *Trauma and traumatic injury* are currently not listed as diagnoses in the DSM-5-TR or ICD-11.
- *Trauma* can be:
  - physical, meaning an injury to living tissue caused by an extrinsic physical, biological, or radiological agent, or
  - psychological, meaning a disordered psychic or behavioural state resulting from severe mental or emotional stress.
- In the mental health context, *trauma* is a person’s experience during an event so distressing that it overwhelms them emotionally. Severe psychological trauma is considered the etiology (or cause) of posttraumatic stress disorder.
- A person can experience physical trauma, for example, a minor laceration, sprain, skin infection, or sunburn, without also experiencing psychological trauma. On the other hand, severe physical trauma that causes unstable multiorgan system polytrauma is often associated with psychological trauma.
- Psychologically stressful experiences are not necessarily traumatic. People can feel stressed without experiencing trauma.
- The word “injury” typically means an acute state, but is often used colloquially to describe a chronic state arising from an acute experience or condition, or a physical or psychological trauma (e.g. an operational stress injury [OSI]).

**Tri-services**

***General public definition***

- *Tri-services* are public safety personnel who are firefighters, paramedics, and police.

***Academic definition***

- *Tri-services* are a subset of public safety personnel that refers specifically to firefighters, paramedics, and police.

**Vicarious trauma**

***General public definition***

- *Vicarious trauma* is currently not listed as a diagnosis in either the DSM-5-TR or ICD-11.
- *Vicarious trauma* is psychological trauma that can occur in people who are indirectly exposed to a potentially psychologically traumatic event (PPTE) (e.g. when providing professional or personal support or care to a traumatized person).
- The term *vicarious trauma* is often used to describe symptoms experienced by a counsellor or therapist who works with patients or clients who have experienced psychologically traumatic events.
- Vicarious trauma is also known as *secondary traumatic stress*.

***Academic definition***

- *Vicarious trauma* is currently not listed as a diagnosis in either the DSM-5-TR or ICD-11.
- *Vicarious trauma* refers to psychological trauma that occurs following indirect exposure to a potentially psychologically traumatic event or exposure to a traumatized person (e.g. learning about another’s trauma, or providing professional or personal support or care to a traumatized person).
- *Vicarious trauma* can be “the transformation that occurs within the therapist as a result of empathic engagement with the client’straumaexperiences and their sequelae.”^71^
- *Vicarious trauma* is believed to be exacerbated by, and perhaps rooted in, the open engagement of empathy, or the connection with the client that is inherent in counselling relationships.^72^,^73^
- *Vicarious trauma* reflects exposure of counsellors to their clients’ accounts of traumatic experiences, and encompasses the subsequent cognitive, emotional, and behavioural disruptions experienced by those counsellors.^72^-^74^

**Vicarious traumatic stress**

***General public definition***

- *Vicarious traumatic stress* is currently not listed as a diagnosis in the DSM-5-TR or ICD-11.
- *Vicarious traumatic stress* is the stress that a person feels when they learn about a potentially psychologically traumatic event experienced by another person.
- Vicarious traumatic stress is also known as *secondary traumatic stress*.
- See also the “Vicarious trauma” section.

***Academic definition***

- *Vicarious traumatic stress* is currently not listed as a diagnosis in either the DSM-5-TR or ICD-11.
- *Vicarious traumatic stress *is the stress that can occur following indirect exposure to a potentially psychologically traumatic event (e.g. learning about another person’s traumatic experience) or exposure to a traumatized person (e.g. when providing professional or personal support or care to a person who has been traumatized).
- Vicarious traumatic stress is also known as *secondary traumatic stress*.
- See also the “Vicarious trauma” section.

**Well-being (wellbeing)**

***General public definition***

- *Well-being*, which can also be written as *wellbeing*, is currently not listed as a diagnosis in the DSM-5-TR or ICD-11.
- Veterans Affairs Canada (VAC) defines *well-being* using a broad framework that includes seven interacting domains:^31^,^34^
  - employment or meaningful purpose
  - financial security
  - health
  - life skills and preparedness
  - social integration
  - housing and physical environment, and
  - cultural and social environment.

***Academic definition***

- *Well-being*, or *wellbeing*, is currently not listed as a diagnosis in either the DSM-5-TR or ICD-11.
- Veterans Affairs Canada (VAC) defines *well-being* using a broad framework that includes seven interacting domains:^31^,^34^
  - employment or meaningful purpose
  - financial security
  - health
  - life skills and preparedness
  - social integration
  - housing and physical environment, and
  - cultural and social environment.
- Elements of each domain in the VAC framework can vary from poor to good, based on subjective and objective measurements.^34^,^35^
- Health is one domain of well-being that interacts with the other domains bidirectionally; for example, having a good job supports good mental health, while having good mental healthsupports finding and keeping a good job.
- The subjective and objective variability from poor to good in each domain underscores the complexity of well-being and the importance of understanding health as part of a system that depends upon, and influences, the other domains of well-being, as opposed to existing in isolation.

**Wellness**

***General public definition***

- *Wellness* is currently not listed as a diagnosis in the DSM-5-TR or ICD-11.
- There is no consensus on a definition of *wellness* at this time. Some definitions of *wellness* overlap with definitions of *well-being*.
- The First Nations Mental Wellness Continuum Framework (FNMWCF) defines *mental wellness* as a balance of the “mental, physical, spiritual, and emotional.”^75^ In this framework, mental wellness is enriched when a person has purpose, hope for their future, a sense of belonging, and a sense of meaning.

***Academic definition***

- *Wellness* is currently not listed as a diagnosis in either the DSM-5-TR or ICD-11.
- There is no consensus on a definition at this time.
- The First Nations Mental Wellness Continuum Framework (FNMWCF) defines *mental wellness* as a balance of the “mental, physical, spiritual, and emotional.”^75^ This balance is enriched when individuals have “purpose in their daily lives whether it is through education, employment, care-giving activities, or cultural ways of being and doing; hope for their future and those of their families that is grounded in a sense of identity, unique Indigenous values, and having a belief in spirit; a sense of belonging and connectedness within their families, to community, and to culture; and finally a sense of meaning and an understanding of how their lives and those of their families and communities are part of creation and a rich history.”^75^
- There is some overlap between the terms, *wellness, health, and well-being*, and at times they are used interchangeably.

**Wellness check**

***General public definition***

- A wellness check is “a [type of call] in which the police are tasked with confirming the well-being of an individual. These calls can be [requested by] family members or the public, or [as a result of] observations made by police officers, and for a variety of different reasons. These calls can also result in a plethora of different outcomes, including confirmation of the individual’s well-being, a referral to community resources, a mental health apprehension, or even an arrest if a crime has occurred.”^76^

***Academic definition***

- “Much ambiguity exists surrounding [the term] ‘wellness check,’ including in [its] definition and application.”^76^ The term has yet to be “formally defined in a widely accepted and standardized manner.”^73^ This gap has resulted in many different interpretations.^76^
- Other terms, which appear to be used interchangeably, also reference the same or similar types of interaction, for example, well-being/wellbeing checks, welfare checks, and check-ins. None of these terms are currently well defined.The terms and associated definitions may also vary from region-to-region and by public safety service or organization.
- “Although not concrete, the term [wellness check] is commonly understood as a type of call in which the police are tasked with confirming the well-being of an individual. These calls can originate from several different sources, including calls from family members or the public, or from observations by police officers, and for a variety of different reasons. These calls can also result in a plethora of different outcomes, including confirmation of the individual’s well-being, a referral to community resources, a mental health apprehension, or even an arrest if a crime has occurred.”76

## Acknowledgements

We thank the following partner organizations (in alphabetical order) for their support in making this resource a reality: the Atlas Institute for Veterans and Families, the Canadian Institute for Military and Veteran Health Research (CIMVHR), the Canadian Institute for Pandemic Health Education and Response (CIPHER), the Canadian Institute for Public Safety Research and Treatment (CIPSRT), Department of National Defence Terminology Board, McMaster University, the Public Health Agency of Canada, Queen’s University, and Veterans Affairs Canada. In addition, we would like to thank our contributors’ employers and universities who allowed their members the time needed to contribute to this glossary.

The senior authors team wishes to acknowledge all the people with lived experience and other stakeholders who dedicated their time and provided thoughtful contributions in collaboratively defining military sexual trauma. 

The senior authors team is grateful to the following individuals for their contributions to the development of this glossary (contributors are listed alphabetically by last name):

LCol (Ret’d) Suzanne Bailey, Curriculum Development Lead, Road to Mental Readiness, Canadian Forces Health Services Group, Department of National Defence

Dr. Mary Bartram, PhD, RSW, Director, Policy, Mental Health Commission of Canada, Adjunct Research Professor, School of Public Policy and Administration, Carleton University

Dr. Suzette Brmault-Phillips, OT, PhD, DCA, Associate Professor, Department of Occupational Therapy, Faculty of Rehabilitation Medicine, University of Alberta; Director, HiMARC (Heroes in Mind, Advocacy and Research Consortium), University of Alberta

Dr. Lori Buchart, PhD, DBA, Co-Founder and Former Co-Chair, It’s Not Just 20K (INJ20K, formerly It’s Not Just 700)

Col Marilynn S. Chenette, CD, CHE, Deputy Commander, Health Services Division (HS Div), Canadian Armed Forces

Dr. Heidi Cramm, PhD, OT Reg. (Ont.), Professor, School of Rehabilitation Therapy, Queen’s University

Department of National Defence Terminology Board

Michelle Douglas, Executive Director, LGBT Purge Fund

Dr. Susan Dowler, PhD, CPsych, Chief Clinical Psychologist, Canadian Forces Health Services Group, Department of National Defence

A/Superintendent Lorraine Downey, BBA, Operations, Peer Support Coordinator, Ottawa Paramedic Service

Gabrielle Dupuis, MSc, Director, Research Partnerships, Atlas Institute for Veterans and Families

Dr. Maya Eichler, PhD, Associate Professor, Political and Canadian Studies, Women’s Studies, Mount Saint Vincent University; Canada Research Chair in Social Innovation and Community Engagement and leader of the Centre for Social Innovation and Community Engagement in Military Affairs, Mount Saint Vincent University

Dr. Murray Enns, MD, FRCPC, Professor, Department of Psychiatry, University of Manitoba; Medical Director and Staff Psychiatrist, Operational Stress Injury Clinic; Staff Psychiatrist, Adult, Health Sciences Centre; Adjunct Scientist, Manitoba Centre for Health Policy

Dr. Kyle Handley, Clinical Psychologist, York Regional Police, Chair of the Canadian Association Chiefs of Police Psychological Services Committee

Dr. Marnin J. Heisel, PhD, CPsych, Professor, Departments of Psychiatry and of Epidemiology & Biostatistics, University of Western Ontario; Scientist, Lawson Health Research Institute

Christine Hutchins, Senior Director, Office of Women and LGBTQ2 Veterans, Veterans Affairs Canada

Dr. Ruth Lanius, MD, PhD, FRCPC, Professor, Department of Psychiatry; Director of Posttraumatic Stress Disorder research unit; Harris-Woodman Chair in Psyche and Soma, Schulich School of Medicine & Dentistry, Western University; Collaborating Clinical Scientist, Homewood Research Institute

Dr. Vivien Lee, PhD, CPsych, Chief Psychologist, Commander, Healthy Workplace Team, Ontario Provincial Police; Clinical Advisor, Boots on the Ground

Sgt. Brent MacIntyre, BA (Hons.) Criminology, BA Sociology, Respect, Ethics and Values, Ottawa Police Service

Dr. Randi McCabe, PhD, CPsych, Professor, Department of Psychiatry & Behavioural Neurosciences, McMaster University; Co-Founder, Hamilton Centre for Cognitive Behavioural Therapy

Dr. Megan McElheran, RPsych, Clinical Psychologist, Wayfound Mental Health Group

Dr. Margaret McKinnon, PhD, CPsych, Homewood Chair in Mental Health and Trauma, Professor and Associate Chair, Research, Department of Psychiatry and Behavioural Neurosciences, McMaster University; Research Lead, Mental Health & Addictions Services, St. Joseph’s Healthcare Hamilton; Senior Scientist, Homewood Research Institute

Dr. Anthony Nazarov, PhD, PMP, Associate Scientific Director, MacDonald Franklin OSI Research Centre; Research Scientist and Adjunct Research Professor, Department of Psychiatry, Schulich School of Medicine & Dentistry, Western University; Adjunct Scientist, Lawson Health Research Institute; Adjunct Research Professor, Department of Psychiatry and Behavioural Neurosciences, McMaster University

Dr. Andrew Nicholson, PhD, Assistant Professor, School of Psychology, University of Ottawa; Director of Clinical Research, Atlas Institute for Veterans and Families

Dr. Deborah Norris, PhD, Professor, Department of Family Studies & Gerontology, Mount Saint Vincent University

Lt Scott Patey, OFS Peer Support Co-Coordinator, Ottawa Fire Services

Alain Pellegroms, Training Coordinator, OFS Medical Program Coordinator, OFS Peer Support Coordinator, Training Division, Ottawa Fire Service

Dr. Jill Price, PhD, Postdoctoral Fellow, Canadian Institute for Public Safety Research and Treatment (CIPSRT); Sessional Professor, Campion College, University of Regina

Dr. Rosemary Ricciardelli, PhD, Professor and Research Chair in Safety, Security, and Wellness, School of Maritimes Studies, Fisheries and Marine Institute, Memorial University of Newfoundland 

Dr. J. Don Richardson, MD, FRCPC, Consultant Psychiatrist, Medical Director, St. Joseph’s Health Care London’s Operational Stress Injury Clinic; Scientific Director, MacDonald Franklin OSI Research Centre; Medical Advisor, Atlas Institute for Veterans and Families; Professor and Wellness Lead, Tanna Schulich Chair in Neuroscience and Mental Health, Schulich School of Medicine & Dentistry, Western University; Associate Scientist, Lawson Health Research Institute 

Dr. Maya Roth, PhD, CPsych, Clinical Psychologist, St. Joseph’s Healthcare London’s Operational Stress Injury Clinic; Affiliated Scientist, MacDonald Franklin OSI Research Centre; Associate Member, Yeates School of Graduate Studies, Toronto Metropolitan University; Adjunct Clinical Professor, Schulich School of Medicine and Dentistry, Western University; Associate Scientist, Lawson Health Research Institute

Martine Roy, Chair, Board of Directors, LGBT Purge Fund

Capt Sam Samplonius, Co-Founder, INJ20K

PC Benny Seto, BBE, Mobile Crisis Intervention Team, Toronto Police Service

Dr. Norman Shields, PhD, CPsych, National Chief Psychologist, Occupational Health and Safety Branch, Royal Canadian Mounted Police

Dr. Lorraine Smith-MacDonald, PhD, MA, MDiv, CCC, Postdoctoral Fellow, Heroes in Mind, Advocacy, and Research Consortium (HiMARC), Faculty of Rehabilitation Medicine, University of Alberta

Dr. Heather Stuart, PhD, FRSC, CM, Professor, Department of Public Health Sciences, Department of Psychiatry and the School of Rehabilitation Therapy, Queen’s University; Bell Canada Chair Mental Health and Anti-Stigma Research, Queen’s University

LCol Dr. Andrea Tuka, CD, MD, FRCPC, Clinical Assistant Professor, Department of Psychiatry, University of British Columbia; Chief of Psychiatry, Canadian Armed Forces 

## Senior authors team

The senior authors team includes the following (listed alphabetically by last name):

Elizabeth Bose, MSc, Knowledge Translation Coordinator, Canadian Institute for Pandemic Health Education and Response (CIPHER)

R. Nicholas Carleton, PhD, RD Psych, Scientific Director, Canadian Institute for Public Safety Research and Treatment (CIPSRT), University of Regina; Professor of Psychology, Department of Psychology, University of Regina

Dr. Dianne Groll, PhD, Adjunct Associate Professor, Departments of Psychiatry and Psychology, Queen’s University

LCol (Ret’d) Dr. Alexandra Heber, FRCPC, Chief of Psychiatry, Veterans Affairs Canada; Executive Director, Canadian Institute for Pandemic Health Education and Response (CIPHER); Associate Professor, Department of Psychiatry and Behavioural Neurosciences, McMaster University 

Yasaman Jabbari, PhD, Research Coordinator, Trauma and Recovery Research Unit, Department of Psychiatry and Behavioural Neurosciences, McMaster University; PhD Student, Cognitive Psychology, Department of Psychology, Neuroscience, and Behaviour, McMaster University

Jillian Lopes, MSc, Clinical Psychology, Department of Psychology, Neuroscience, and Behaviour, McMaster University; Research Assistant, Trauma and Recovery Research Unit, Department of Psychiatry and Behavioural Neurosciences, McMaster University

Ashlee Mulligan, MSc, Director, Partnerships and Stakeholder Engagement, Atlas Institute for Veterans and Families, The Royal

Dr. Kimberley Ritchie, PhD, MN, BScN, Assistant Professor, Trent/Fleming School of Nursing, Trent University; Adjunct Professor, Department of Psychiatry and Behavioural Neurosciences, McMaster University

Amber Schick, MA, Research Associate, Canadian Institute for Pandemic Health Education and Response (CIPHER)

Emily Sullo, MMASc, BSc (Hons), Research Assistant, Trauma and Recovery Unit, Department of Psychiatry and Behavioural Neurosciences, McMaster University; PhD Student, Clinical Psychology, Department of Psychology, Neuroscience, and Behaviour, McMaster University 

Dr. Linna Tam-Seto, PhD, OTReg (Ont.), Assistant Professor, Department of Occupational Science and Occupational Therapy, Temerty Faculty of Medicine, University of Toronto

Valerie Testa, MSc, BEd, BA (Hons), OCT, CCRP, Senior Policy Analyst, Trauma Supports and Wellbeing Unit, Prevention Division, Centre for Mental Health and Wellbeing, Public Health Agency of Canada

## Statement

The content and views expressed in this article are those of the authors and do not necessarily reflect those of the Government of Canada.
