# Corrigendum - Glossary of terms: A shared understanding of the common terms used to describe psychological trauma, version 3.0

**DOI:** 10.24095/hpcdp.44.4.06

**Published:** 2024-04

This corrigendum is being published to remove two bullets from a definition in the following article: 

Heber A, Testa V, Groll D, Ritchie K, Tam-Seto L, Mulligan A, Sullo E, Schick A, Bose E, Jabbari Y, Lopes J, Carleton RN. Glossary of terms: A shared understanding of the common terms used to describe psychological trauma, version 3.0. Health Promot Chronic Dis Prev Can. 2023;43(10/11). https://doi.org/10.24095/hpcdp.43.10/11.09

The first two bullets have been removed from the Military sexual trauma (MST) definition. 

At the time of this publication, the Department of National Defence (DND) Terminology Board endorsed the definition of Military Sexual Trauma (MST) provided. Although published on DND’s website on September 26, 2023, this definition was removed from its website in early January 2024. 

## Before correction

***Military sexual trauma (MST)***

**General public and academic definition**

- Military sexual trauma (MST) is trauma caused to a Canadian Armed Forces (CAF) member as a result of unwanted sexual or sexualized activity by another CAF member. There can be varying degrees and impacts of trauma.
- (The above is the official definition endorsed by the Department of National Defence Terminology Board and the opening sentence of the Glossary of Terms 3.0 definition.)
- MST is currently not listed as a diagnosis in the DSM-5-TR or ICD-11.
- MST refers to any sexual or sexualized activity that occurs without the person’s consent, during their service as a member of the CAF, and the physically or psychologically traumatic impacts of this activity on the affected person. The spectrum of MST can vary from small impact to severe disorders.
- Examples of sexual or sexualized activities without the person’s consent or where the person is unable to consent include (but are not limited to):

- Taking part in sexual activities because of coercion or threat (such as threats to a person’s physical safety, reputation, or career progression, or threats of other negative treatment, if the person refuses to comply)
- Any coercive situation where expectation of, participation in, or tolerance of, unwanted sexual experiences is used as a basis for work assignment or promotion decisions
- Any situation involving comments, unwanted touching, grabbing, or sexual advances, including hazing activities or rituals
- Sexual contact or activities while sleeping, unconscious, or any other circumstance where the person’s capacity to consent is impaired by drugs or alcohol
- Sexualized comments or displays of pornographic or demeaning materials in the workplace
- Repeated unwelcome requests for a sexual relationship
- Witnessing any of the examples of sexual or sexualized activities in this list
- Any unwanted sexual activity or display that creates a hostile, intimidating, or offensive work environment.

- Examples of MST impacts on the affected person include (but are not limited to):

- Disturbed sleep or nightmares
- Feeling sad or depressed
- Disturbing memories of re-experiencing the event
- Difficulty feeling safe
- Feeling numb or without emotion
- Feeling guilt or shame, anger or rage
- Problems in work (such as reduced productivity, conflict with coworkers)
- Problems in intimate relationships, and difficulties parenting
- Problems with alcohol or drugs
- Physical injuries or pain conditions, and
- Reluctance to report for duty or to wear their uniform.

## After correction

***Military sexual trauma (MST)***

**General public and academic definition**

- MST is currently not listed as a diagnosis in the DSM-5-TR or ICD-11.
- MST refers to any sexual or sexualized activity that occurs without the person’s consent, during their service as a member of the CAF, and the physically or psychologically traumatic impacts of this activity on the affected person. The spectrum of MST can vary from small impact to severe disorders.
- Examples of sexual or sexualized activities without the person’s consent or where the person is unable to consent include (but are not limited to):

- Taking part in sexual activities because of coercion or threat (such as threats to a person’s physical safety, reputation, or career progression, or threats of other negative treatment, if the person refuses to comply)
- Any coercive situation where expectation of, participation in, or tolerance of, unwanted sexual experiences is used as a basis for work assignment or promotion decisions
- Any situation involving comments, unwanted touching, grabbing, or sexual advances, including hazing activities or rituals
- Sexual contact or activities while sleeping, unconscious, or any other circumstance where the person’s capacity to consent is impaired by drugs or alcohol
- Sexualized comments or displays of pornographic or demeaning materials in the workplace
- Repeated unwelcome requests for a sexual relationship
- Witnessing any of the examples of sexual or sexualized activities in this list
- Any unwanted sexual activity or display that creates a hostile, intimidating, or offensive work environment.

- Examples of MST impacts on the affected person include (but are not limited to):

- Disturbed sleep or nightmares
- Feeling sad or depressed
- Disturbing memories of re-experiencing the event
- Difficulty feeling safe
- Feeling numb or without emotion
- Feeling guilt or shame, anger or rage
- Problems in work (such as reduced productivity, conflict with coworkers)
- Problems in intimate relationships, and difficulties parenting
- Problems with alcohol or drugs
- Physical injuries or pain conditions, and
- Reluctance to report for duty or to wear their uniform.

The original online version of the article has been modified on February 28, 2024, to reflect this change. Once available, the PDF version of the article will reflect the updated version of the definition.

